# Tau in neurodegenerative diseases: molecular mechanisms, biomarkers, and therapeutic strategies

**DOI:** 10.1186/s40035-024-00429-6

**Published:** 2024-08-06

**Authors:** Xingyu Zhang, Jiangyu Wang, Zhentao Zhang, Keqiang Ye

**Affiliations:** 1 Department of Neurology, Renmin Hospital of Wuhan University, Wuhan, 430060 China; 2 Taikang Center for Life and Medical Sciences, Wuhan University, Wuhan, 430000 China; 3 Faculty of Life and Health Sciences, Shenzhen Institute of Advanced Technology, Chinese Academy of Sciences, Shenzhen, 518055 China

**Keywords:** Tau, Alzheimer’s disease, Tauopathies, Aggregation, Propagation, Biomarkers

## Abstract

The deposition of abnormal tau protein is characteristic of Alzheimer’s disease (AD) and a class of neurodegenerative diseases called tauopathies. Physiologically, tau maintains an intrinsically disordered structure and plays diverse roles in neurons. Pathologically, tau undergoes abnormal post-translational modifications and forms oligomers or fibrous aggregates in tauopathies. In this review, we briefly introduce several tauopathies and discuss the mechanisms mediating tau aggregation and propagation. We also describe the toxicity of tau pathology. Finally, we explore the early diagnostic biomarkers and treatments targeting tau. Although some encouraging results have been achieved in animal experiments and preclinical studies, there is still no cure for tauopathies. More in-depth basic and clinical research on the pathogenesis of tauopathies is necessary.

## Introduction

Tau is a microtubule-associated protein encoded by the *MAPT* gene, which is located on chromosome 17q21 and contains 16 exons [1]. Tau can be subdivided into the N-terminal projection domain and the C-terminal assembly domain. The N-terminal projection domain sticks away from microtubules, while the C-terminal assembly domain contains the repeat domains and flanking regions that bind to microtubules and contribute to the aggregation of tau. Between amino acids 151 and 243, several Thr-Pro and Ser-Pro motifs can be phosphorylated [2]. There are six main isoforms of tau expressed in the adult human brain. The variants vary based on the 29-residue inserts near the amino terminus: the variants with 0, 1, or 2 inserts are referred to as 0N, 1N, and 2N, respectively. The isoforms can also be classified according to the inclusion of three or four repeat domains, known as 3R or 4R, respectively [1]. In the brain, tau is predominantly expressed in neurons, while glial cells contain only trace amounts of tau [3]. Furthermore, tau has been detected extracellularly [4]. The cellular localization of tau is controlled by developmental processes [5]. In immature neurons, tau is evenly distributed in the cell body and cell extensions. Later, as axons emerge and neurons become polarized, tau is enriched in axons, with small quantities present in dendrites and the nuclei [6, 7].

Physiologically, tau plays diverse roles in various parts of neurons. In healthy neurons, tau in axons regulates the stability of microtubules and microtubule dynamics, and influences axonal transport [8]. A small quantity of tau is also present in dendrites, where it may play a role in mediating excitotoxicity and regulating synaptic plasticity, post-synaptic microtubule dynamics, and neuronal physiology [9]. Tau in the nucleus may contribute to the preservation of genomic DNA integrity [7, 10]. Recent studies have revealed various abnormal changes in tau knockout mice, suggesting that tau might be involved in the control of neurogenesis, neuronal function, and long-term depression [1].

Pathologically, tau abnormalities are implicated in various tauopathies, including Alzheimer’s disease (AD), progressive supranuclear palsy (PSP), frontotemporal dementia with parkinsonism-17 (FTDP-17), corticobasal degeneration (CBD), chronic traumatic encephalopathy (CTE), argyrophilic grain disease (AGD), Pick’s disease, and Huntington’s disease. Over 80 mutations of the *MAPT* gene have been discovered to be associated with tauopathies [11, 12]. In this review, we extensively discuss the role of tau in neurodegenerative diseases, with a focus on the mechanisms of tau aggregation or propagation, the toxicity of tau pathology, and early diagnostic biomarkers and treatments targeting tau.

## Tau pathology in neurodegenerative diseases

The term "tauopathy" was initially described as a specific condition known as frontotemporal lobar degeneration (FTLD) [13]. Soon after, the term "tauopathies" was adopted to encompass a diverse array of degenerative disorders affecting the nervous system, both sporadic and hereditary. These disorders are characterized by the presence of tangled accumulations of hyperphosphorylated tau, which can be found in neurons or both neurons and glial cells. Tauopathies encompass more than 20 distinct neurodegenerative disorders and can be further divided into primary and secondary tauopathies. In primary tauopathies such as PSP, FTDP-17, CBD, Pick’s disease, and AGD, tau pathology is considered the primary cause of neurodegeneration, whereas in secondary tauopathies such as AD and CTE, tau pathology is considered as having diverse driving force [14].

Pick’s disease predominantly features three-repeat (3R) tau aggregates with round, well-defined inclusions in the neuronal cytoplasm. In contrast, PSP and CBD present diverse neuronal and glial inclusions composed of four-repeat (4R) tau. CTE is characterized by clusters of twisted neurofibrillary tangles (NFTs) composed of both 3R and 4R tau, noticeable tangled fibers within the neuropil, and astroglial tau pathology [15]. In AD, a mix of 3R and 4R tau aggregates exists in the affected brain area. These observations support the concept that the presence of different pathogenic tau strains may cause distinct tauopathy subtypes.

### AD

AD is a progressive neurodegenerative disease and is the most common cause of dementia worldwide. Clinically, AD is characterized by gradual declines in memory and cognitive function, and difficulties in daily tasks [16]. Neuropathologically, AD is characterized by the presence of amyloid plaques and NFTs [17]. NFTs are primarily composed of truncated and aberrantly hyperphosphorylated tau, forming paired helical filaments (PHFs) or straight filaments. Importantly, the density and distribution of NFTs are consistently related to the extent of brain atrophy, cognitive decline, and memory impairment [18–20], suggesting that tau pathology may play a pivotal role in the development of dementia in AD patients.

### FTLD

FTLD is a group of neurodegenerative diseases characterized by atrophy of the frontal and temporal lobes [21]. FTLD is one of the most common causes of dementia, only next to AD and dementia with Lewy bodies (DLB). FTLD is characterized by changes in social behavior and personality, speech and language. FTLD can occur either independently or in conjunction with motor disorders [22]. Neuropathologically, FTLD is characterized by the presence of aberrant protein aggregates formed by tau, TDP-43, or FUS (fused in sarcoma protein) [23]. Nearly 95% of clinical FTD cases are FTLD-tau and FTLD-TDP.

### PSP

PSP, a type of neurodegenerative disease, was initially described by Steele and his team in 1964 [24]. It typically starts after the age of 40 and has an average survival time of six years from the onset of symptoms. Clinically, individuals with PSP exhibit impairments in motor skills, gait control, balance, swallowing, speech production, and vision. Patients also display symptoms of parkinsonism, sensitivity to light, sleep and emotional disturbances, depression, anxiety, and dementia [23, 24].

Neuropathologically, PSP is characterized by the presence of tufted astrocytes, accompanied by the existence of NFTs and threads within the basal ganglia and brainstem [25]. While the proportion of 3R and 4R isoforms in the healthy brain is 1:1, protein aggregates formed by 4R tau are found in patients with PSP [26]. Injecting brain extracts from patients with PSP into tau transgenic mice can recapitulate PSP-like tau inclusions, suggesting the presence of template-dependent amplification of tau aggregates [27].

### CBD

CBD refers to a neurodegenerative disease characterized by pathological tau deposition in various cell types and anatomical regions [28]. Clinically, the symptoms of CBD include behavioral, dysexecutive, and visuospatial syndromes; nonfluent, agrammatic primary progressive aphasia syndrome; and progressive supranuclear palsy-like syndrome. Neuropathologically, CBD is characterized by the presence of a glial pathology that encompasses hyperphosphorylated 4R tau [28]. Other tau-related abnormalities are also found in the CBD, including neuronal inclusions, threads, and coiled bodies [28–30].

### CTE

CTE is a disease caused by long-term exposure to repeated hits to the head. Symptoms of CTE include memory impairment, cognitive confusion, compromised decision-making, difficulties in controlling impulses, aggressive behavior, depression, anxiety, suicidal thoughts, parkinsonism, and, ultimately, progressive dementia. The manifestation of these symptoms is often delayed by years or even decades following the occurrence of the last head injury or the cessation of active sports engagement [31]. Neuropathologically, CTE is characterized by the accumulation of phosphorylated tau, the formation of NFTs, astrocytic tangles, and neurites around small blood vessels in the cortex, which are typically located at the depths of the sulci. Severe cases of CTE display widespread tau pathology throughout the entire brain [32]. Most individuals with CTE exhibit abnormal levels of phosphorylated TDP-43. Additionally, amyloid-β (Aβ) aggregates are found in 43% of cases. The tau isoform profile and phosphorylation state in CTE resemble those observed in AD, which involve both 3R and 4R tau [33].

### Tau pathology in synucleinopathies

Synucleinopathies refer to a group of neurodegenerative diseases in which abnormal accumulation of misfolded α-synuclein protein occurs, resulting in diseases such as Parkinson's disease (PD), DLB, and multiple system atrophy (MSA) [34–36]. Tauopathies and synucleinopathies exhibit overlapping clinical, pathological, and genetic features. Clinically, cognitive decline and parkinsonism are frequently observed in PD, DLB, MSA, PSP, and CBD patients. Pathologically, tau pathology may also manifest as Lewy bodies and glial cytoplasmic inclusions within synucleinopathies. Furthermore, the presence of *MAPT* H1 haplotype is associated with an increased risk of synucleinopathies such as PD and potentially DLB and MSA [37, 38]. These shared characteristics between synucleinopathies and tauopathies suggest the possibility of interactions or common pathways contributing to neurodegeneration.

## The mechanisms mediating tau aggregation

### Posttranslational modifications (PTMs) of tau

Tau undergoes various PTMs, including phosphorylation, acetylation, methylation, ubiquitination, SUMOylation, DOPAGEL, and truncation. These PTMs may affect the function and aggregation propensity of tau by altering its charge, hydrophobicity, and structural properties.

#### Tau phosphorylation

Phosphorylation is the most common PTM of tau and usually involves three types of amino acids: serine (S), threonine (T) and tyrosine (Y). 2N4R tau has 85 potential phosphorylation sites [39]. Tau phosphorylation is regulated by various kinases and phosphatases. The kinases are divided into three groups: (1) protein kinases PDPKs (proline-directed protein kinases), including GSK3β, CDK5, and MAPKs; (2) protein kinases non-PDPKs, including TTBK1/2, CK1a/1e/2, PKA, PKB/Akt, PKC, PKN, and CaMKII; and (3) tyrosine kinases, including Src, Lck, Syk, Fyn, and c-Abl kinase [39]. Several tau kinases, such as GSK3β, CDK5, DYRK1A, and CK1, have been reported to be involved in the abnormal phosphorylation of tau in the AD brain [39–42]. The dephosphorylation of tau is mediated by phosphatases such as protein phosphatase (PP)-1, PP5, PP2A, PP2B, and PTEN (phosphatase and tensin homolog deleted on chromosome 10) [43]. The activity or expression of these phosphatases is disrupted in AD brains [44].

Hyperphosphorylation of tau may lead to the dissociation of tau from microtubules and hamper its capacity to facilitate microtubule polymerization [45]. Despite extensive research, the impact of phosphorylation on tau aggregation remains controversial. It has been proposed that phosphorylation of S214, S258, S262, S293, S305, S324, and S356 inhibits tau aggregation [45–47], while phosphorylation of T149, T153, S199, S202, T205, and T212 increases aggregation [48–50]. Biophysical characterization has shown that phosphorylation regulates the aggregation propensity of tau by neutralizing charge interactions and inducing conformational transitions, including disruption of local turn and β-sheet structures, formation of transient α-helices, opening of transient folds, and reorganization of the dimer interface [51–55].

#### Acetylation

Tau lysine acetylation is mediated by the p300/CREB binding protein, HAT, and tau itself [56–60]. In patients with early and moderate Braak stages of tauopathy, tau acetylation levels are elevated and contribute to neurotoxicity, neuronal dysfunction, and cognitive decline [61]. Tau acetylation reduces the binding of tau to microtubules, impairs its ability to promote tubulin assembly, and affects its degradation [61, 62].

The impact of acetylation on tau pathological aggregation also remains controversial. On the one hand, acetylation can induce the formation of β-sheet structures, thereby accelerating tau filament assembly [63, 64]. However, other studies have shown that acetylation inhibits tau aggregation [56, 65, 66]. Thus, the exact roles of tau acetylation under physiological and pathological conditions warrant further study.

#### Methylation

Tau has been reported to undergo mono- and dimethylation at 11 sites [67]. Methylated tau is widely present in AD brains and colocalizes with NFTs in late-stage AD [68]. Methylation has minimal effects on the affinity of tau for microtubules and on tau-mediated tubulin polymerization. Notably, the aggregation propensity of methylated tau is dramatically attenuated during both the nucleation and elongation steps, suggesting that lysine methylation protects tau from aggregation [67].

#### Ubiquitination and SUMOylation

Tau has a high propensity for ubiquitination [69]. The ubiquitination of tau enhances its clearance through the proteasome or lysosomal autophagy system [68–70]. However, ubiquitinated tau oligomers may impair proteasome function [71]. Furthermore, ubiquitination also inhibits tau-mediated microtubule assembly [72] and promotes tau aggregation [69]. Recently, Arakhamia et al. reported that the ubiquitination of tau may help stabilize interprotofilament packing and regulate polymorphisms in tauopathies [73].

SUMOylation is mainly related to protein interactions and subcellular localization and usually occurs on lysine residues. For tau, SUMOylation targets K340R via small ubiquitin-like modifier 1 (SUMO1) [74, 75]. The SUMOylation of tau is closely associated with its phosphorylation. SUMO1 is colocalized with phosphorylated tau in the cerebral cortex of the AD brain. Tau SUMOylation promotes tau hyperphosphorylation. Additionally, tau hyperphosphorylation stimulates its SUMOylation. In addition, tau SUMOylation inhibits tau degradation by decreasing the solubility and ubiquitination of tau [76]. Some animal experiments have suggested that SUMOylation may be related to age [77]. These results suggest that the ubiquitination and SUMOylation of tau may play a key role in the development of tau pathology.

#### Truncation

In the AD brain, tau is abnormally truncated by many proteases. Tau is cleaved by caspase 2 at Asp314, by caspase 3 at Asp25 and Asp421, by caspase 6 at Asp13 and Asp402, by chymotrypsin at Tyr197, by calpain at Lys44 and Arg230 [78], and by asparaginyl endopeptidase (AEP) at Asn255 and Asn368 [79]. Among these proteases, the activity of AEP in the brain increases in an age-dependent manner. AEP cleaves tau at residues Asn255 and Asn368, generating a tau (1-368) fragment that is more prone to phosphorylation and aggregation than full-length tau [79]. AEP deletion in tau P301S transgenic mice reduces tau pathology and ameliorates cognitive deficits. The tau (1-368) fragment enhances BACE1 expression and Aβ production, propagating AD pathology [80]. Furthermore, compound #11, a small-molecule inhibitor of AEP, attenuates AD pathology and partially rescues cognitive deficits in mouse models of AD [81].

#### 3,4-Dihydroxyphenylacetaldehyde (DOPEGAL) modification

DOPEGAL is the aldehyde metabolite of norepinephrine. Accumulation of the catecholamine-derived aldehyde DOPEGAL within neurons is thought to be one of the mechanisms triggering neurodegeneration in AD [82]. In AD patients, the concentration of DOPEGAL in the locus coeruleus is elevated by approximately three folds [82]. Recently, it was reported that DOPEGAL activates AEP and subsequently cleaves tau, promoting tau aggregation in AD [83]. Furthermore, DOPEGAL covalently modifies the Lys353 residue of tau in the locus coeruleus and triggers tau aggregation, accelerating its pathology and spreading to interconnected brain regions [84].

### Cross-seeding of tau with other prion-like proteins and cryo-EM structural analysis of different tau strains

#### Assembly model for tau

The aggregation of tau follows a nucleation–elongation mechanism. The primary processes include dimerization, corresponding to filament nucleation (the rate-limiting nucleation event), and elongation (the addition of monomers to the ends of growing polymers). The second processes include filament fragmentation (increasing the number of filament ends available for elongation), secondary nucleation (producing small, highly diffusible aggregates associated with toxicity), and filament annealing [85]. The "nucleation phase" proceeds slowly with unfavorable thermodynamics, whereas the "elongation phase" proceeds rapidly with more favorable thermodynamics. Therefore, the kinetics of tau assembly display a sigmoidal growth curve with a lag phase followed by a rapid growth phase and a final plateau phase. The rate-limiting step in aggregation is the assembly of misfolded proteins into nuclei (seeds). Amyloid formation can be significantly accelerated by adding preformed seeds. The seeds can reduce the lag time and promote amyloid formation by transforming normal proteins into fibrils (seeding).

#### Cross-seeding of tau with other prion-like proteins

Several studies have documented cross-seeding between tau and other misfolded proteins, including Aβ, α-synuclein, and IAPP.

The most representative cross-seeding studies in animal models involve Aβ and tau. The simultaneous accumulation of these proteins in the brain is a major hallmark of AD. Aβ has been found to accelerate tau aggregation, while tau aggregates do not have the same effect on Aβ [86, 87]. In human studies, advances in molecular positron emission tomography (PET) have enabled tracking of tau pathology and Aβ pathology in AD. A PET imaging study identified some converging areas of Aβ and tau pathology, particularly in the inferolateral temporal lobe, suggesting a physical interaction of these pathologies in disease progression [88]. Another PET imaging study revealed that tau pathology begins focally but progresses rapidly under the influence of Aβ pathology [89]. The interaction between Aβ and tau is linked to neurodegeneration and cognitive decline. These results indicate the synergistic role of Aβ and tau in the pathogenesis of AD.

In a subgroup of AD patients, tau and α-synuclein pathologies occur simultaneously. In addition, multiple studies have shown that tau and α-synuclein aggregate in PD and DLB brains [90–92]. These results suggest that α-syn and tau may synergistically promote fibrillation of each other. Tau interacts with α-syn directly via the microtubule-binding region of the tau protein and the C-terminus of α-syn [93]. This binding allows cross-seeding between tau and α-syn. In vitro, co-incubation of α-syn and tau synergistically promotes the aggregation of both proteins [94]. In vivo, α-syn preformed fibrils (PFFs) were shown to induce tau aggregation in cultured cells and in the brain [95–98]. On the other hand, tau PFFs also promote the aggregation and spread of α-syn in PD [99]. These results suggest that tau and α-syn accelerate the seeding and spreading of each other.

Patients with type 2 diabetes mellitus (T2DM) have an increased incidence of AD [100]. T2DM is characterized by the deposition of islet amyloid polypeptide (IAPP) in the pancreas [101]. IAPP interacts with tau to accelerate the formation of a more virulent strain that exhibits enhanced seeding activity and neurotoxicity. Intrahippocampal injection of tau fibrils formed in the presence of IAPP, into tau P301S transgenic mice, triggered the spread of tau pathology, synaptic loss, and cognitive deficits [102].

#### Cryo-EM structural analysis of different tau strains

The aggregation of tau is similar to that of prions, which transform from a soluble monomeric state to a state of self-propagating aggregates rich in β-sheet structures [103]. Prions adopt pathological conformations called "strains" to stably propagate in living systems and create unique neuropathological patterns. Data from multiple studies suggest that tau functions as a prion [104–106]. Diamond and colleagues isolated tau strains from 29 patients with 5 different tauopathies and found that different diseases are linked to distinct sets of strains [105]. Recent breakthroughs in electron cryo-microscopy have allowed the atomic structure of tau filaments to be extracted from the brains of individuals with various tauopathies. The characteristics of each disease are unique tau filament folding, which remains conserved among individuals suffering from the same disease [107].

In the brains of AD patients, tau inclusions have two types of cryo-EM structures, PHF and straight filaments, which consist of a common C-shaped ordered core [108]. PHFs and straight filaments are distinguished by different interprotofilament packing arrangements. The ordered core contains 306–378 amino acids (numbered by the 441-amino-acid tau isoform). The remaining amino acids at the N- and C-termini adopt random conformations termed the fuzzy coat [109]. Additional cases of AD or different brain regions from individual cases of AD show the same tau filament structures [110]. Since valine 306 at the beginning of the ordered core is the first amino acid of R3, 4R tau or 3R tau monomers can both be incorporated into filaments, which explains the presence of 3R and 4R tau PFFs in AD. If the ordered core includes amino acids containing R1 followed by R3 or R2 followed by R3, then such structures can recruit 3R or 4R tau isoforms, respectively [108].

In the past several years, the structure of tau fibrils in Pick's disease [111], CTE [112], CBD [28], PSP, and other tauopathies [113] has been determined by cryo-EM. Most R1s and no R2s are present in the ordered cores of Pick's disease filaments, explaining their selectivity for 3R tau. R2s are present in the ordered cores of tau filaments in CBD, PSP, and other diseases, explaining why they contain only 4R tau.

## The mechanisms mediating tau propagation and spreading

### Transmission of tau pathology

During the progression of AD, tau pathology typically begins in the brainstem, including the locus coeruleus (LC), and then progresses to the transentorhinal cortex or the entorhinal cortex in the medial temporal lobe (Braak stages I and II), then to the hippocampal region (Braak stages III and IV), and finally to the neocortex or the primary areas of the neocortex (Braak stages V and VI) [114]. An increasing number of studies have suggested that the LC may be the origin of tau pathology [114–116]. Jacobs et al. performed human-level neuroimaging research, including magnetic resonance imaging (MRI) measurements of LC integrity and tau PET imaging, and found that changes in LC integrity precede tau accumulation in the medial temporal lobe. In addition, the selective vulnerability of the LC to tau is related to specific genetic features [117]. Tau protein spreads in the brain through neuronal connections, which may involve multiple mechanisms, including spreading between strongly interacting brain regions (functional connectivity), through anatomical connections (structural connectivity), or simple diffusion. A study using magnetoencephalography/PET revealed that structural connections and functional connections play important roles in tau transmission[118].

In FTLD-Tau, this apparent aspect of tau diffusion is less well characterized. In PSP, an initial pallido‐luyso‐nigral stage of tau deposition, progressing to the basal ganglia, the pontine and dentate nuclei, and the frontal and parietal lobe, has been reported [119]. In AGD, the initial sites of deposition are the ambient gyrus and its vicinity, the anterior and posterior medial temporal lobe, and the septum, insular cortex, and anterior cingulate gyrus, accompanied by spongy degeneration of the ambient gyrus [120]. These observations support the notion that pathological forms of tau spread between neurons and that the roots of tau transmission are distinct in different tauopathies.

### Mechanisms that mediate neuron–neuron transmission of tau

Neuron-neuron transmission of tau is believed to be mediated by exocytosis and endocytosis. Exocytosis mechanisms include exosome release, secretion, and neuronal death. Wang et al. demonstrated that neurons can release tau via exosomes and that neuronal activity enhances the release of exosomes [121]. Notably, exosome-associated tau levels have been reported to be elevated in the cerebrospinal fluid (CSF) and blood of patients with AD and FTD [122, 123]. These findings suggest that exosomal processes are involved in the cell-to-cell spread of tau. Endocytosis mechanisms include micropinocytosis, phagocytosis, dynamin-mediated endocytosis, receptor-mediated endocytosis, and/or membrane fusion of exosomes. Macropinocytosis-mediated tau internalization in neurons is mediated by heparan sulfate proteoglycans (HSPGs) [124, 125], which bind to extracellular tau aggregates and promote their cellular uptake [126]. Low-density lipoprotein receptor-related protein 1 acts synergistically with HSPGs to control tau entry into neurons [127], but its contribution to tau pathogenesis has not been determined. In addition, the dynamin-dependent endocytosis pathway also regulates tau endocytosis [128]. *BIN1*/Amphiphyrin2, a genetic risk factor for late-onset AD, has also been shown to regulate clathrin-mediated endocytosis of pathological tau aggregates [129]. Another important mechanism mediating tau endocytosis is receptor-mediated endocytosis. One study screened for receptors of pathological tau and revealed that receptor for advanced glycation end products (RAGE) mediates neuronal uptake of pathological forms of tau [130]. RAGE deficiency reduces transsynaptic tau spread and inhibits microglial inflammatory responses. Furthermore, RAGE is needed for tau-induced memory loss, while blocking the interaction between RAGE and tau oligomers ameliorates cognitive impairment in rTg4510 mice [130]. These results suggest that RAGE plays an important role in tau pathogenesis. In addition, M1/M3 muscarinic receptors have also been reported to mediate tau uptake [131]. The muscarinic receptor antagonists atropine and pirenzepine block 80% of this uptake.

### Microglia in the spread of tau pathology

Several studies have reported that microglia phagocytose free tau [132–135]. Microglial HSPGs, CX3CR1 and P2RY12 have been shown to bind tau [136–138]. Multiple mechanisms are involved in tau uptake by microglia, including phagocytosis, macropinocytosis, and micropinocytosis [139]. Microglia may release tau seeds through intracellular tau-mediated microglial cytotoxicity, resulting in the release of tau seeds that have not yet been degraded [140]. In addition, microglia can secrete tau through exosomes, which may be a way for microglia to directly transmit tau [141, 142]. Blocking exosome release or depleting microglia mitigates the spread of tau in a tau-inoculated mouse model [133]. In addition, microglia secrete factors that may exacerbate the spread of tau in neurons [143, 144]. Several studies have shown that microglia are able to degrade tau [132, 145, 146]. Thus, microglia may mediate the clearance of tau, but in advanced tauopathies, the degradation capacity of microglia may be overwhelmed by tau aggregates and the secretion of tau fibrils with enhanced seeding activity.

## The toxicity of pathological tau

The presence of pathological tau undoubtedly triggers a series of cellular dysfunctions, including mitochondrial dysfunction, endoplasmic reticulum stress, cytoskeletal instability, synaptic dysfunction, and disruption of axon transport, and eventually leads to neurodegeneration [147–151]. Tau is present in various forms, ranging from soluble monomers to insoluble fibrils. Insoluble NFTs are considered classical toxic and pathogenic entities that parallel the duration and severity of the disease [152–154]. However, this perspective has been challenged in recent years. Some studies have shown that certain soluble forms of tau may play a more important role [155, 156]. Tau oligomers impair memory consolidation by inducing synaptic and mitochondrial dysfunction in wild-type mice [157]. Reducing soluble tau levels partially reversed cognitive impairments in animal models [158]. Tau oligomers also damage neuronal nuclei, impairing nucleocytoplasmic transport and altering pathogenic gene expression [159]. Compared with tau fibrils, tau oligomers have not been the subject of much research. In the future, more research is needed to explore the toxicity and pathogenic mechanism of tau oligomers.

Notably, tau not only exerts neurotoxicity but also indirectly affects other neurodegenerative proteins, such as Aβ, α-synuclein and IAPP [160]. The interaction between tau and other prion-like proteins may regulate the toxicity of tau aggregates [160–162]. Clinical studies have revealed greater cognitive decline in older adults with concurrent abnormalities in CSF Aβ and p-tau [163, 164]. Furthermore, Aβ and tau interactions in the inferotemporal neocortex exacerbate tau pathology and cognitive decline. Individuals with hypometabolism in the posterior cingulate gyrus, where tau–Aβ interactions are most closely related, show progressive memory decline [165]. These results suggest that synergy between Aβ and tau is associated with cognitive decline and brain dysfunction. Several studies have shown the synergistic effect of Aβ and tau on microglia, astrocytes, or organelles such as mitochondria[166, 167]. The copathology of α-syn and tau also promotes the pathological spreading of proteins more strongly than does the administration of tau or α-syn alone[168]. Administration of α-syn/tau oligomers derived from PD patients to the brains of tau transgenic mice accelerated the formation of tau oligomers and induced more severe neuronal loss than did administration of tau oligomers alone [169]. In addition, injection of tau/α-syn mixed fibrils exacerbates the spread of tau pathology in a mouse model of tauopathy compared with injection of pure tau or α-syn fibrils[170]. The mixed fibrils of IAPP and tau also exhibit enhanced seeding activity and neurotoxicity both in vitro and in vivo. Compared with tau fibrils, intrahippocampal injection of IAPP-tau mixed strains into tau P301S transgenic mice significantly promoted the spread of tau pathology and induced more severe synaptic loss and cognitive deficits [102]. Thus, the synergistic effect of tau and other prion proteins deserves further study.

As we described previously, tau PTMs can also enhance tau toxicity. Hyperphosphorylation of tau can lead to mislocalization of tau to the somatogenic compartment, reduce microtubule binding, and promote tau misfolding [171]. Tau acetylation impairs microtubule binding, reduces solubility, promotes cleavage, and impairs protein degradation[172–174]. The truncation of tau also affects tau toxicity by promoting tau aggregation, reducing microtubule binding, and promoting synaptic dysfunction[175].

## Pathological tau as a diagnostic biomarker

Due to the crucial role of tau in the occurrence and development of this disease, a diagnosis based on tau pathology is urgently needed. At present, the diagnosis of pathological tau mainly includes CSF-based biomarkers, blood-based biomarkers, and tau-PET images [176].

### CSF-based tau biomarkers

The elevation of p-tau in the CSF signifies the presence of pathology. For the past few decades, p-tau181 in the CSF has been found to be one of the core biomarkers of AD. Recently, the diagnostic capabilities of tau phosphorylated at other sites have begun to be recognized. It appears that p-tau217 is superior to p-tau181 as a CSF biomarker for the differential diagnosis of AD [177–179]. Recently, p-tau205 was also identified as a biomarker for AD [180]. Another study revealed that MTBR-tau243 is particularly specific to tau aggregates and is strongly associated with tau PET [181]. In addition to tau phosphorylation and aggregation, fragmentation of tau also plays a role in tau pathogenesis. AEP cleaves tau, thereby generating the tau (1-368) fragment, which is increased in patients with AD [79]. Blennow et al. showed that tau368 is a tangle-enriched fragment. The tau368/total-tau (t-tau) ratio in the CSF was decreased in patients with AD and negatively correlated with ^18^F-GTP1 retention [182]. In addition, they compared the levels of CSF p-tau 181, p-tau217, t-tau, and tau368 and their correlation with tau burden in cognitively unimpaired, mild cognitive impairment, non-AD cognitive disorder, and AD dementia patients. In symptomatic AD patients, tau368/t-tau was more strongly associated with tau-PET scanning and cognitive performance than other CSF tau biomarkers [183]. Although CSF-based biomarkers show high accuracy in the diagnosis of AD, blood-based biomarkers are needed because lumber puncture is invasive.

### Blood-based tau biomarkers

Blood-based tau biomarkers are more economical, more accessible and less invasive. Among them, p-tau181, p-tau217 and p-tau231 are the most promising [184–188]. The plasma level of phosphorylated tau is correlated with the density of Aβ and tau [189–192]. Additionally, plasma markers can effectively differentiate AD from other neurodegenerative diseases and have certain predictive power for the future development of AD [188, 193]. Notably, p-tau217 performs slightly better than others, possibly because of its higher levels in AD [190, 194]. The blood p-tau217 test is comparable or superior to the CSF p-tau217 test in the detection of AD pathology [195, 196]. Recent research has shown that the diagnostic accuracy of p-tau212 in blood is similar to that of p-tau217 [197]. In addition, blood-based brain-derived tau has been identified as a biomarker for identifying Aβ-positive individuals at risk of short-term cognitive decline and atrophy [198]. The development of blood-based biomarkers will aid in the early noninvasive diagnosis and treatment of tauopathy.

### PET imaging of tau pathology

Tau-PET tracers are used to visualize tau aggregates and identify the distribution and stage of tau pathology. The first tau-PET tracer, [^18^F]-flortaucipir, was approved by the U.S. Food and Drug Administration (FDA) in May 2020 for the clinical detection of AD. This tracer has good blood-brain barrier (BBB) permeability and excellent metabolism. This tracer is less sensitive to tau associated with tauopathies other than AD [199]. Second-generation tau-PET tracers, including [^18^F]-MK6240, [^18^F]-RO948, and [^18^F]-PI2620, can more selectively bind to hippocampal tau. Tau-PET is predictive of cognitive disorders in AD patients [200]. Furthermore, the findings of tau-PET perfusion and [^18^F]-FDG-PET metabolism are in strong agreement [201]. Recently, Isla et al. compared three tracers: [^18^F]-Flortaucipir, [^18^F]-MK-6240 and [^18^F]-PI-2620. The three tracers showed similar autoradiographic binding characteristics. They all bind strongly to NFTs in AD but do not significantly bind to tau aggregates in non-AD tauopathies, suggesting their limited utility in detecting non-AD tauopathies in vivo. None of them bind to Aβ, α-synuclein, or TDP-43 lesions, but they all bind strongly to neuromelanin and melanophore-containing cells and weakly to hemorrhage areas [202]. The off-target effects of PET tracers need to be further improved, and non-AD tauopathy PET tracers are also worthy of further study. Compared to fluid biomarkers, tau-PET is more accurate at the expense of high cost. Each of these approaches has advantages and disadvantages. It might be practical to use blood-based biomarkers as a screening index and then choose CSF and even tau-PET for further diagnosis.

## Therapeutic strategies against tau pathology

### Targeting tau production and aggregation

**Fig. 1:**
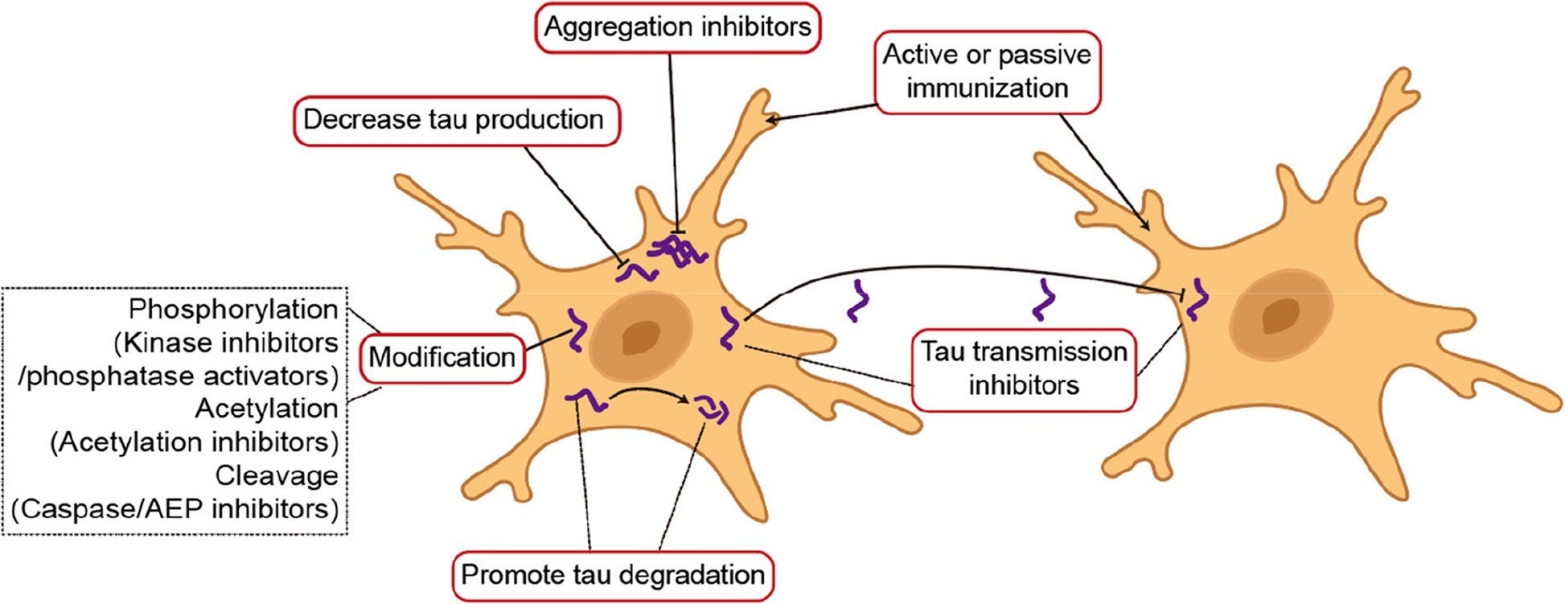
Therapeutic strategies against tau pathology, including (1) inhibiting tau production, such as by siRNAs and ASOs; (2) inhibiting tau aggregation, such as the methylene blue derivatives LMTX and curcumin; (3) regulating the post-translational modifications of tau, such as kinase inhibitors/phosphatase activators, acetylation inhibitors, and caspase/AEP inhibitors; (4) promoting tau degradation, such as autophagy or proteasomal degradation regulators; (5) inhibiting tau transmission; and (6) active and passive immunotherapies

Targeting tau production is a way to inhibit tau pathology (Fig. 1). Cellular and animal experiments have shown that siRNAs targeting tau reduce tau pathology and neurodegeneration in tau P301S transgenic mice [203]. Intrathecal injection of antisense oligonucleotides (ASOs) targeting tau decreased the mRNA expression and protein level of tau in cynomolgus monkeys. The reduction of tau was observed in the frontal cortex, temporal cortex and hippocampus, indicating good transmission in the brain [204]. The results from a phase Ib trial (NCT03186989) indicated that the tau ASO MAPT Rx (also known as BIIB080) is safe and reduces t-tau and p-tau levels in the CSF of patients with mild AD [205]. Phase II trials have also been initiated in patients with MCI and AD (NCT05399888). A group of tau aggregation inhibitors has also been identified [206]. Methylene blue (MB) hinders the aggregation of tau [207, 208]. A customized intranasal hydrogel delivering MB was developed to ameliorate cognitive dysfunction [209]. LMTX (TRx0237) is a derivative of MB. TRx0237 is currently being evaluated in the LUCIDITY Phase III trial. The interim results showed that the improvements in the treatment group were less than expected. The natural product curcumin also binds to β-sheets and prevents aggregation [210]. Recently, Wang et al. identified a set of aptamer candidates, including BW1c, which has a high binding affinity for tau and significantly inhibits tau oligomerization and aggregation [211]. In addition, Yao et al. synthesized a group of isatin-pyrrolidinylpyridine compounds that inhibit tau self-aggregation and even depolymerize tau aggregates [212]. These promising compounds deserve further clinical study.

### Targeting tau degradation and transmission

Promoting the degradation of tau is another therapeutic strategy (Fig. 1). Tau is cleared through the ubiquitin-proteasome system, the endosomal–lysosomal system, and the autophagy-lysosome system. The phosphorylation of 26S proteasomes induced by cAMP/PKA promotes the degradation of tau [213]. Some phosphodiesterase inhibitors are currently undergoing clinical trials [214]. Anti-tau scFv chimeras were developed to direct tau to the proteasome or lysosome, reducing intracellular tau levels [215]. Tau-targeting proteolysis-targeting chimeras (PROTACs), which can lead to polyubiquitination and proteasome-mediated degradation of the protein, were developed to reduce t-tau and p-tau levels in tauopathy mouse models [216, 217]. Lysosome-targeting chimeras and antibody-based PROTACs were also designed to promote lysosomal targeting and clearance of tau[218, 219]. The autophagy-targeting chimera complex was developed to clear tau through the autophagy–lysosomal system [220]. Recently, a study revealed pathogenic tau-specific autophagy based on a customized nanochaperone that enhanced autophagic flux and pathological tau clearance, alleviating the tau burden and cognitive deficits in AD mice [221]. However, this strategy requires further clinical validation. There are currently several antibodies designed to prevent the transmission of tau, such as BIIB076 [222], Tilavonemab, and PRX005. Nevertheless, further clinical studies are needed to validate these antibodies in the future.

### Targeting tau modification

Several drugs have been developed to target tau hyperphosphorylation, including lithium [223, 224], phosphatase modifiers, and synthetic small molecules [225, 226]. Lithium is an inhibitor of GSK3-β [227]. The long-term administration of lithium reduces amyloid plaque formation, reduces tau hyperphosphorylation, and improves learning and memory in transgenic mice that overproduce Aβ and tau [227–229]. However, the cognitive outcomes did not improve in a phase 2 clinical trial [230]. Another phase II trial (NCT02862210) to assess the effects of lithium on the behavioral symptoms of FTD was completed, but the results have yet to be reported. Sodium selenite has been shown to reduce tau phosphorylation in animal models [231, 232]. However, in a clinical trial of patients with mild to moderate AD, diffusion MR images were slightly improved. In a phase Ib open-label study (ACTRN12617001218381) in patients with FTD, MRI and cognitive and behavioral measures were slightly reduced. Two phase IIb trials in patients with FTD (ACTRN12620000236998) and PSP (ACTRN12620001254987) are ongoing. Salsalate and diflunisal were shown to reduce tau acetylation by inhibiting p300 acetyltransferase [233]. Pieper et al. reported that patients receiving salsalate or diflunisal exhibited a decreased incidence of AD [61]. However, a phase I open-label study (NCT02422485) in PSP patients showed that the drug was well tolerated but failed to improve disease progression in PSP patients [234]. A second phase I trial (NCT03277573) involving AD patients has been completed, but the results have not yet been reported. Minocycline and VX-765 are caspase inhibitors that also play a positive role in AD [235–237]. A multicenter phase II study (ISRCTN16105064) of minocycline in AD patients showed that minocycline increased adverse effects and failed to slow disease progression. The AEP inhibitor compound 11 reduced tau cleavage and resulted in the amelioration of tau pathology and protection of cognitive function in tau P301S and 5×FAD transgenic mice [81]. However, further clinical research on compound 11 is needed.

### Tau immunotherapies

Immunotherapies have achieved remarkable progress in recent years. Active immunization involves the induction of antigen-antibody reactions with low doses of tau fragments. The initial attempt at active immunization was to treat C57 mice with full-length human tau protein, which resulted in various side effects, including axonal damage and encephalomyelitis. Researchers subsequently turned to tau fragments [206, 238]. To date, active immunization therapies including AADvac1 (targeting phosphorylation-independent conformational epitope) and ACI-35 (targeting Ser396/Ser404 epitope) are in clinical trials [239–242]. Four clinical studies of AADvac1 have been completed. In a phase I trial (NCT01850238), AADvac1 was shown to be safe and well tolerated in AD patients [239]. A follow-up phase I study (NCT02031198, FUNDAMANT) showed a similar safety profile, and higher IgG titers were significantly associated with reduced hippocampal atrophy and cognitive decline [241]. The results from a phase II trial (NCT02579252, ADAMANT) showed that AADvac1 was safe and well tolerated and induced a strong IgG response [240]. The vaccine did not alter cognition or brain atrophy rates but was associated with a 58% attenuation of plasma NfL increases. However, larger stratified studies are still needed to evaluate the clinical effectiveness of this vaccine. An open-label phase I pilot trial (NCT03174886) was conducted in patients with nonfluent, agrammatic variant progressive aphasia (naPPA). The results have not yet been reported. A phase Ib study (ISRCTN13033912) revealed that ACI-35 was safe and well tolerated but had a weak immune response in patients with mild to moderate AD. The second-generation vaccine ACI-35.030 has improved immunogenicity and produces antibodies that specifically bind p-tau and recognize PHF in the brains of AD patients. A phase Ib/IIa trial (NCT04445831) is ongoing to test the safety and immunogenicity of ACI-35.030 in early AD. Interim results showed that all groups generated specific and potent antibody responses against p-tau and PHF and that there were no clinically relevant safety concerns [243]. Passive immunity is the administration of tau antibodies to target extracellular or intracellular tau to block its prion-like seeding [244, 245]. Multiple clinical trials have attempted to use tau antibodies to treat tauopathies [246, 247]. A phase I study (NCT05344989) of APNmAb005 (an anti-tau IgG antibody) is expected to be completed in July 2024. Three phase I trials of Bepranemab (UCB0107) (an IgG4 antibody that binds to aa 235–250 of tau) (NCT03464227, NCT03605082, and NCT04185415) showed that UCB0107 was safe and that the level of UCB0107 in CSF increased in a dose-dependent manner. A phase II trial of UCB0107 (NCT04867616) is ongoing. In addition, many tau antibodies, such as BIIB076, E2814, gosuranemab, and JNJ-63733657, are undergoing clinical trials. However, many trials have been terminated due to safety concerns.

### Challenges and future opportunities of tau treatment

Currently, treatments for tau are in their infancy. Although many drug candidates targeting tau have been discovered and developed, few are clinically useful. Tau treatment faces several challenges. (1) The BBB limits the entry of drugs. More strategies need to be developed to pass through the BBB. (2) The mechanism of tauopathy remains unclear. Many issues need to be further addressed in the future. What initiates tauopathy? What is the relationship between Aβ pathology and tau pathology? Is tau the cause or a byproduct of tauopathy? Which form of tau exerts toxic effects? What is the relationship between different tau strains and clinical manifestations? To develop new therapeutic strategies, we need to further explore the cellular and molecular pathways involved in the pathophysiology of tauopathies. (3) Animal or cellular models of tau pathology do not fully mimic human disease progression, and the results observed in vitro are difficult to replicate in clinical trials. We need to further develop novel tauopathy models, such as 3D organoids [248] and human iPSC tauopathy models [249]. (4) The timing of clinical intervention remains questionable. In clinical trials, clinical intervention may be too late to reverse disease progression. The development of new biomarkers is needed to facilitate early diagnosis. Furthermore, it may be more important to develop biomarkers in blood than in CSF. (5) Some clinical trials had a small number of participants or had a short observation period. In the future, larger clinical trials are needed, recruiting a larger number of patients, observing for a longer period, and designing more innovative and efficient clinical trials. In addition, further clinical trials of combination therapies including tau and Aβ are needed.

## Conclusions

Here we have discussed the clinical and neuropathological features of different tauopathies. Tau plays an important role in these neurodegenerative diseases. Converging lines of evidence support that tau acts as a prion-like protein. Various post-translational modifications affect its aggregation. However, the effects of some post-translational modifications, such as tau phosphorylation and acetylation, on aggregation are controversial and deserve further investigation. The spread of tau is mediated by tau exocytosis and endocytosis, in which microglia play a mysterious role. Tau has several conformational states, including monomers, oligomers, and fibrils. Among them, oligomers are considered to be the most toxic. The pathogenic mechanisms of these different conformational states of tau also deserve more in-depth studies. CSF-, blood- and PET-based tau biomarkers are useful for the diagnosis of AD. Multiple approaches targeting tau have been explored for the treatment of AD and other tauopathies. Although animal experiments and preclinical studies have achieved encouraging results, the clinical data are not optimistic. Therefore, more in-depth basic research on tauopathy is necessary to determine the exact role of tau in neurodegenerative disease and to identify new therapeutic targets.

## Data Availability

Not applicable.

## Abbreviations

AD: Alzheimer’s disease
PSP: Progressive supranuclear palsy
FTDP-17: Frontotemporal dementia with parkinsonism-17
CBD: Corticobasal degeneration
CTE: Chronic traumatic encephalopathy
AGD: Argyrophilic grain disease
NFTs: Neurofibrillary tangles
PHFs: Paired helical filaments
DLB: Dementia with Lewy bodies
Aβ: Amyloid-β
PD: Parkinson's disease
MSA: Multiple system atrophy
PTMs: Post-translational modifications
PP: Protein phosphatase
SUMO1: Small ubiquitin-like modifier 1
AEP: Asparaginyl endopeptidase
DOPEGAL: 3,4-Dihydroxyphenylacetaldehyde
PFF: Preformed fibril
T2DM: Type 2 diabetes mellitus
IAPP: Islet amyloid polypeptide
HSPG: Heparan sulfate proteoglycan
RAGE: Advanced glycation end products
ASO: Antisense oligonucleotide
MB: Methylene blue

## References

[1] Wang Y, Mandelkow E. Tau in physiology and pathology. Nat Rev Neurosci. 2016;17:5–21. 26631930 10.1038/nrn.2015.1

[2] Hanger DP, Anderton BH, Noble W. Tau phosphorylation: the therapeutic challenge for neurodegenerative disease. Trends Mol Med. 2009;15:112–9. 19246243 10.1016/j.molmed.2009.01.003

[3] Kahlson MA, Colodner KJ. Glial tau pathology in tauopathies: functional consequences. J Exp Neurosci. 2015;9:43–50. 26884683 10.4137/JEN.S25515 PMC4750898

[4] Yamada K. Extracellular tau and its potential role in the propagation of tau pathology. Front Neurosci. 2017;11:667. 29238289 10.3389/fnins.2017.00667 PMC5712583

[5] Drubin DG, Caput D, Kirschner MW. Studies on the expression of the microtubule-associated protein, tau, during mouse brain development, with newly isolated complementary DNA probes. J Cell Biol. 1984;98:1090–7. 6421824 10.1083/jcb.98.3.1090 PMC2113151

[6] Papasozomenos SC, Binder LI. Phosphorylation determines two distinct species of Tau in the central nervous system. Cell Motil Cytoskeleton. 1987;8:210–26. 2446784 10.1002/cm.970080303

[7] Sultan A, Nesslany F, Violet M, Bégard S, Loyens A, Talahari S, et al. Nuclear tau, a key player in neuronal DNA protection. J Biol Chem. 2011;286:4566–75. 21131359 10.1074/jbc.M110.199976 PMC3039398

[8] Barbier P, Zejneli O, Martinho M, Lasorsa A, Belle V, Smet-Nocca C, et al. Role of tau as a microtubule-associated protein: structural and functional aspects. Front Aging Neurosci. 2019;11:204. 31447664 10.3389/fnagi.2019.00204 PMC6692637

[9] Ittner A, Ittner LM. Dendritic tau in Alzheimer’s disease. Neuron. 2018;99:13–27. 30001506 10.1016/j.neuron.2018.06.003

[10] Violet M, Delattre L, Tardivel M, Sultan A, Chauderlier A, Caillierez R, et al. A major role for Tau in neuronal DNA and RNA protection in vivo under physiological and hyperthermic conditions. Front Cell Neurosci. 2014;8:84. 24672431 10.3389/fncel.2014.00084 PMC3957276

[11] Kouri N, Carlomagno Y, Baker M, Liesinger AM, Caselli RJ, Wszolek ZK, et al. Novel mutation in MAPT exon 13 (p.N410H) causes corticobasal degeneration. Acta Neuropathol. 2014;127:271–82. 24121548 10.1007/s00401-013-1193-7 PMC3943649

[12] Coppola G, Chinnathambi S, Lee JJ, Dombroski BA, Baker MC, Soto-Ortolaza AI, et al. Evidence for a role of the rare p.A152T variant in MAPT in increasing the risk for FTD-spectrum and Alzheimer’s diseases. Hum Mol Genet. 2012;21:3500–12. 22556362 10.1093/hmg/dds161 PMC3392107

[13] Spillantini MG, Goedert M, Crowther RA, Murrell JR, Farlow MR, Ghetti B. Familial multiple system tauopathy with presenile dementia: a disease with abundant neuronal and glial tau filaments. Proc Natl Acad Sci U S A. 1997;94:4113–8. 9108114 10.1073/pnas.94.8.4113 PMC20577

[14] Kovacs GG. Molecular pathological classification of neurodegenerative diseases: turning towards precision medicine. Int J Mol Sci. 2016;17:189. 26848654 10.3390/ijms17020189 PMC4783923

[15] Grossman M, Seeley WW, Boxer AL, Hillis AE, Knopman DS, Ljubenov PA, et al. Frontotemporal lobar degeneration. Nat Rev Dis Primers. 2023;9:40. 37563165 10.1038/s41572-023-00447-0

[16] 2023 Alzheimer’s disease facts and figures. Alzheimers Dement. 2023;19:1598–695 10.1002/alz.13016 36918389

[17] Zhang W, Wang H-F, Kuo K, Wang L, Li Y, Yu J, et al. Contribution of Alzheimer’s disease pathology to biological and clinical progression: A longitudinal study across two cohorts. Alzheimers Dement. 2023;19:3602–12. 36840615 10.1002/alz.12992

[18] Giannakopoulos P, Herrmann FR, Bussière T, Bouras C, Kövari E, Perl DP, et al. Tangle and neuron numbers, but not amyloid load, predict cognitive status in Alzheimer’s disease. Neurology. 2003;60:1495–500. 12743238 10.1212/01.WNL.0000063311.58879.01

[19] Smirnov DS, Salmon DP, Galasko D, Goodwill VS, Hansen LA, Zhao Y, et al. Association of neurofibrillary tangle distribution with age at onset-related clinical heterogeneity in Alzheimer disease: an autopsy study. Neurology. 2022;98:e506-17. 34810247 10.1212/WNL.0000000000013107 PMC8826459

[20] Fonseca CS, Baker SL, Dobyns L, Janabi M, Jagust WJ, Harrison TM. Tau accumulation and atrophy predict amyloid independent cognitive decline in aging. Alzheimers Dement. 2024;20:2526–37. 38334195 10.1002/alz.13654 PMC11032527

[21] Cairns NJ, Bigio EH, Mackenzie IRA, Neumann M, Lee VM-Y, Hatanpaa KJ, et al. Neuropathologic diagnostic and nosologic criteria for frontotemporal lobar degeneration: consensus of the Consortium for Frontotemporal Lobar Degeneration. Acta Neuropathol. 2007;114:5–22. 17579875 10.1007/s00401-007-0237-2 PMC2827877

[22] Boeve BF, Boxer AL, Kumfor F, Pijnenburg Y, Rohrer JD. Advances and controversies in frontotemporal dementia: diagnosis, biomarkers, and therapeutic considerations. Lancet Neurol. 2022;21:258–72. 35182511 10.1016/S1474-4422(21)00341-0

[23] Kwiatkowski TJ, Bosco DA, Leclerc AL, Tamrazian E, Vanderburg CR, Russ C, et al. Mutations in the FUS/TLS gene on chromosome 16 cause familial amyotrophic lateral sclerosis. Science. 2009;323:1205–8. 19251627 10.1126/science.1166066

[24] Steele JC, Richardson JC, Olszewski J. Progressive supranuclear palsy: a heterogeneous degeneration involving the brain stem, basal ganglia and cerebellum with vertical gaze and pseudobulbar palsy, nuchal dystonia and dementia. Arch Neurol. 1964;10:333–59. 14107684 10.1001/archneur.1964.00460160003001

[25] Grimm M-J, Respondek G, Stamelou M, Arzberger T, Ferguson L, Gelpi E, et al. Clinical conditions “suggestive of progressive supranuclear palsy”-diagnostic performance. Mov Disord. 2020;35:2301–13. 32914550 10.1002/mds.28263 PMC7953080

[26] Rösler TW, Tayaranian Marvian A, Brendel M, Nykänen N-P, Höllerhage M, Schwarz SC, et al. Four-repeat tauopathies. Prog Neurobiol. 2019;180: 101644. 31238088 10.1016/j.pneurobio.2019.101644

[27] Clavaguera F, Akatsu H, Fraser G, Crowther RA, Frank S, Hench J, et al. Brain homogenates from human tauopathies induce tau inclusions in mouse brain. Proc Natl Acad Sci U S A. 2013;110:9535–40. 23690619 10.1073/pnas.1301175110 PMC3677441

[28] Zhang W, Tarutani A, Newell KL, Murzin AG, Matsubara T, Falcon B, et al. Novel tau filament fold in corticobasal degeneration. Nature. 2020;580:283–7. 32050258 10.1038/s41586-020-2043-0 PMC7148158

[29] Murray ME, Kouri N, Lin W-L, Jack CR, Dickson DW, Vemuri P. Clinicopathologic assessment and imaging of tauopathies in neurodegenerative dementias. Alzheimers Res Ther. 2014;6:1. 24382028 10.1186/alzrt231 PMC3978456

[30] Koga S, Josephs KA, Aiba I, Yoshida M, Dickson DW. Neuropathology and emerging biomarkers in corticobasal syndrome. J Neurol Neurosurg Psychiatry. 2022;93:919–29. 35697501 10.1136/jnnp-2021-328586 PMC9380481

[31] McKee AC, Stein TD, Huber BR, Crary JF, Bieniek K, Dickson D, et al. Chronic traumatic encephalopathy (CTE): criteria for neuropathological diagnosis and relationship to repetitive head impacts. Acta Neuropathol. 2023;145:371–94. 36759368 10.1007/s00401-023-02540-w PMC10020327

[32] McKee AC, Stein TD, Kiernan PT, Alvarez VE. The neuropathology of chronic traumatic encephalopathy. Brain Pathol. 2015;25:350–64. 25904048 10.1111/bpa.12248 PMC4526170

[33] Schmidt ML, Zhukareva V, Newell KL, Lee VM, Trojanowski JQ. Tau isoform profile and phosphorylation state in dementia pugilistica recapitulate Alzheimer’s disease. Acta Neuropathol. 2001;101:518–24. 11484824 10.1007/s004010000330

[34] Dickson DW, Braak H, Duda JE, Duyckaerts C, Gasser T, Halliday GM, et al. Neuropathological assessment of Parkinson’s disease: refining the diagnostic criteria. Lancet Neurol. 2009;8:1150–7. 19909913 10.1016/S1474-4422(09)70238-8

[35] Koga S, Sekiya H, Kondru N, Ross OA, Dickson DW. Neuropathology and molecular diagnosis of synucleinopathies. Mol Neurodegener. 2021;16:83. 34922583 10.1186/s13024-021-00501-z PMC8684287

[36] Stefanova N, Bücke P, Duerr S, Wenning GK. Multiple system atrophy: an update. Lancet Neurol. 2009;8:1172–8. 19909915 10.1016/S1474-4422(09)70288-1

[37] Zhang C-C, Zhu J-X, Wan Y, Tan L, Wang H-F, Yu J-T, et al. Meta-analysis of the association between variants in MAPT and neurodegenerative diseases. Oncotarget. 2017;8:44994–5007. 28402959 10.18632/oncotarget.16690 PMC5546535

[38] Li J, Ruskey JA, Arnulf I, Dauvilliers Y, Hu MTM, Högl B, et al. Full sequencing and haplotype analysis of MAPT in Parkinson’s disease and rapid eye movement sleep behavior disorder. Mov Disord. 2018;33:1016–20. 29756641 10.1002/mds.27385

[39] Martin L, Latypova X, Terro F. Post-translational modifications of tau protein: implications for Alzheimer’s disease. Neurochem Int. 2011;58:458–71. 21215781 10.1016/j.neuint.2010.12.023

[40] Engmann O, Giese KP. Crosstalk between Cdk5 and GSK3beta: Implications for Alzheimer’s Disease. Front Mol Neurosci. 2009;2:2. 19521544 10.3389/neuro.02.002.2009 PMC2694676

[41] Branca C, Shaw DM, Belfiore R, Gokhale V, Shaw AY, Foley C, et al. Dyrk1 inhibition improves Alzheimer’s disease-like pathology. Aging Cell. 2017;16:1146–54. 28779511 10.1111/acel.12648 PMC5595697

[42] Roth A, Sander A, Oswald MS, Gärtner F, Knippschild U, Bischof J. Comprehensive characterization of CK1δ-mediated tau phosphorylation in Alzheimer’s disease. Front Mol Biosci. 2022;9:872171. 36203870 10.3389/fmolb.2022.872171 PMC9531328

[43] Martin L, Latypova X, Wilson CM, Magnaudeix A, Perrin M-L, Terro F. Tau protein phosphatases in Alzheimer’s disease: the leading role of PP2A. Ageing Res Rev. 2013;12:39–49. 22771380 10.1016/j.arr.2012.06.008

[44] Chung S-H. Aberrant phosphorylation in the pathogenesis of Alzheimer’s disease. BMB Rep. 2009;42:467–74. 19712581 10.5483/BMBRep.2009.42.8.467

[45] Haj-Yahya M, Gopinath P, Rajasekhar K, Mirbaha H, Diamond MI, Lashuel HA. Site-specific hyperphosphorylation inhibits, rather than promotes, tau fibrillization, seeding capacity, and its microtubule binding. Angew Chem Int Ed Engl. 2020;59:4059–67. 31863676 10.1002/anie.201913001 PMC7065254

[46] Kumar S, Tepper K, Kaniyappan S, Biernat J, Wegmann S, Mandelkow E-M, et al. Stages and conformations of the Tau repeat domain during aggregation and its effect on neuronal toxicity. J Biol Chem. 2014;289:20318–32. 24825901 10.1074/jbc.M114.554725 PMC4106345

[47] Strang KH, Sorrentino ZA, Riffe CJ, Gorion K-MM, Vijayaraghavan N, Golde TE, et al. Phosphorylation of serine 305 in tau inhibits aggregation. Neurosci Lett. 2019;692:187–92. 30423399 10.1016/j.neulet.2018.11.011 PMC6351168

[48] Bailey RM, Covy JP, Melrose HL, Rousseau L, Watkinson R, Knight J, et al. LRRK2 phosphorylates novel tau epitopes and promotes tauopathy. Acta Neuropathol. 2013;126:809–27. 24113872 10.1007/s00401-013-1188-4 PMC3830748

[49] Chang E, Kim S, Schafer KN, Kuret J. Pseudophosphorylation of tau protein directly modulates its aggregation kinetics. Biochim Biophys Acta. 2011;1814:388–95. 20974297 10.1016/j.bbapap.2010.10.005 PMC3018534

[50] Necula M, Kuret J. Pseudophosphorylation and glycation of tau protein enhance but do not trigger fibrillization in vitro. J Biol Chem. 2004;279:49694–703. 15364924 10.1074/jbc.M405527200

[51] Chen D, Drombosky KW, Hou Z, Sari L, Kashmer OM, Ryder BD, et al. Tau local structure shields an amyloid-forming motif and controls aggregation propensity. Nat Commun. 2019;10:2493. 31175300 10.1038/s41467-019-10355-1 PMC6555816

[52] Despres C, Byrne C, Qi H, Cantrelle F-X, Huvent I, Chambraud B, et al. Identification of the Tau phosphorylation pattern that drives its aggregation. Proc Natl Acad Sci U S A. 2017;114:9080–5. 28784767 10.1073/pnas.1708448114 PMC5576827

[53] Rani L, Mittal J, Mallajosyula SS. Effect of phosphorylation and O-GlcNAcylation on proline-rich domains of tau. J Phys Chem B. 2020;124:1909–18. 32065850 10.1021/acs.jpcb.9b11720 PMC7459333

[54] Cantrelle FX, Loyens A, Trivelli X, Reimann O, Despres C, Gandhi NS, et al. Phosphorylation and O-GlcNAcylation of the PHF-1 epitope of tau protein induce local conformational changes of the C-terminus and modulate tau self-assembly into fibrillar aggregates. Front Mol Neurosci. 2021;14:661368. 34220449 10.3389/fnmol.2021.661368 PMC8249575

[55] Rani L, Mallajosyula SS. Phosphorylation-induced structural reorganization in tau-paired helical filaments. ACS Chem Neurosci. 2021;12:1621–31. 33877805 10.1021/acschemneuro.1c00084

[56] Cohen TJ, Friedmann D, Hwang AW, Marmorstein R, Lee VMY. The microtubule-associated tau protein has intrinsic acetyltransferase activity. Nat Struct Mol Biol. 2013;20:756–62. 23624859 10.1038/nsmb.2555 PMC3827724

[57] Cook C, Carlomagno Y, Gendron TF, Dunmore J, Scheffel K, Stetler C, et al. Acetylation of the KXGS motifs in tau is a critical determinant in modulation of tau aggregation and clearance. Hum Mol Genet. 2014;23:104–16. 23962722 10.1093/hmg/ddt402 PMC3857946

[58] Kamah A, Huvent I, Cantrelle F-X, Qi H, Lippens G, Landrieu I, et al. Nuclear magnetic resonance analysis of the acetylation pattern of the neuronal Tau protein. Biochemistry. 2014;53:3020–32. 24708343 10.1021/bi500006v

[59] Min S-W, Cho S-H, Zhou Y, Schroeder S, Haroutunian V, Seeley WW, et al. Acetylation of tau inhibits its degradation and contributes to tauopathy. Neuron. 2010;67:953–66. 20869593 10.1016/j.neuron.2010.08.044 PMC3035103

[60] Tseng J-H, Ajit A, Tabassum Z, Patel N, Tian X, Chen Y, et al. Tau seeds are subject to aberrant modifications resulting in distinct signatures. Cell Rep. 2021;35:109037. 33910013 10.1016/j.celrep.2021.109037 PMC8135111

[61] Shin M-K, Vázquez-Rosa E, Koh Y, Dhar M, Chaubey K, Cintrón-Pérez CJ, et al. Reducing acetylated tau is neuroprotective in brain injury. Cell. 2021;184:2715-2732.e23. 33852912 10.1016/j.cell.2021.03.032 PMC8491234

[62] Xia Y, Bell BM, Giasson BI. Tau K321/K353 pseudoacetylation within KXGS motifs regulates tau-microtubule interactions and inhibits aggregation. Sci Rep. 2021;11:17069. 34426645 10.1038/s41598-021-96627-7 PMC8382713

[63] Haj-Yahya M, Lashuel HA. Protein semisynthesis provides access to tau disease-associated post-translational modifications (PTMs) and paves the way to deciphering the tau PTM code in health and diseased states. J Am Chem Soc. 2018;140:6611–21. 29684271 10.1021/jacs.8b02668

[64] Trzeciakiewicz H, Tseng J-H, Wander CM, Madden V, Tripathy A, Yuan C-X, et al. A dual pathogenic mechanism links tau acetylation to sporadic tauopathy. Sci Rep. 2017;7:44102. 28287136 10.1038/srep44102 PMC5347034

[65] Cohen TJ, Guo JL, Hurtado DE, Kwong LK, Mills IP, Trojanowski JQ, et al. The acetylation of tau inhibits its function and promotes pathological tau aggregation. Nat Commun. 2011;2:252. 21427723 10.1038/ncomms1255 PMC3120096

[66] Ferreon JC, Jain A, Choi K-J, Tsoi PS, MacKenzie KR, Jung SY, et al. Acetylation disfavors tau phase separation. Int J Mol Sci. 2018;19:1360. 29734651 10.3390/ijms19051360 PMC5983838

[67] Funk KE, Thomas SN, Schafer KN, Cooper GL, Liao Z, Clark DJ, et al. Lysine methylation is an endogenous post-translational modification of tau protein in human brain and a modulator of aggregation propensity. Biochem J. 2014;462:77–88. 24869773 10.1042/BJ20140372 PMC4292886

[68] Thomas SN, Funk KE, Wan Y, Liao Z, Davies P, Kuret J, et al. Dual modification of Alzheimer’s disease PHF-tau protein by lysine methylation and ubiquitylation: a mass spectrometry approach. Acta Neuropathol. 2012;123:105–17. 22033876 10.1007/s00401-011-0893-0 PMC3249157

[69] Kim JH, Lee J, Choi WH, Park S, Park SH, Lee JH, et al. CHIP-mediated hyperubiquitylation of tau promotes its self-assembly into the insoluble tau filaments. Chem Sci. 2021;12:5599–610. 34168795 10.1039/D1SC00586C PMC8179656

[70] Chu T-T, Gao N, Li Q-Q, Chen P-G, Yang X-F, Chen Y-X, et al. Specific knockdown of endogenous tau protein by peptide-directed ubiquitin-proteasome degradation. Cell Chem Biol. 2016;23:453–61. 27105281 10.1016/j.chembiol.2016.02.016

[71] Myeku N, Clelland CL, Emrani S, Kukushkin NV, Yu WH, Goldberg AL, et al. Tau-driven 26S proteasome impairment and cognitive dysfunction can be prevented early in disease by activating cAMP-PKA signaling. Nat Med. 2016;22:46–53. 26692334 10.1038/nm.4011 PMC4787271

[72] Munari F, Barracchia CG, Parolini F, Tira R, Bubacco L, Assfalg M, et al. Semisynthetic modification of tau protein with di-ubiquitin chains for aggregation studies. Int J Mol Sci. 2020;21:4400. 32575755 10.3390/ijms21124400 PMC7352214

[73] Arakhamia T, Lee CE, Carlomagno Y, Duong DM, Kundinger SR, Wang K, et al. Posttranslational modifications mediate the structural diversity of tauopathy strains. Cell. 2020;180:633-644.e12. 32032505 10.1016/j.cell.2020.01.027 PMC7491959

[74] Dorval V, Fraser PE. Small ubiquitin-like modifier (SUMO) modification of natively unfolded proteins tau and alpha-synuclein. J Biol Chem. 2006;281:9919–24. 16464864 10.1074/jbc.M510127200

[75] Takamura H, Nakayama Y, Ito H, Katayama T, Fraser PE, Matsuzaki S. SUMO1 modification of tau in progressive supranuclear palsy. Mol Neurobiol. 2022;59:4419–35. 35567706 10.1007/s12035-022-02734-5 PMC9167224

[76] Luo H-B, Xia Y-Y, Shu X-J, Liu Z-C, Feng Y, Liu X-H, et al. SUMOylation at K340 inhibits tau degradation through deregulating its phosphorylation and ubiquitination. Proc Natl Acad Sci U S A. 2014;111:16586–91. 25378699 10.1073/pnas.1417548111 PMC4246270

[77] Nisticò R, Ferraina C, Marconi V, Blandini F, Negri L, Egebjerg J, et al. Age-related changes of protein SUMOylation balance in the AβPP Tg2576 mouse model of Alzheimer’s disease. Front Pharmacol. 2014;5:63. 24778618 10.3389/fphar.2014.00063 PMC3985012

[78] Gu J, Xu W, Jin N, Li L, Zhou Y, Chu D, et al. Truncation of Tau selectively facilitates its pathological activities. J Biol Chem. 2020;295:13812–28. 32737201 10.1074/jbc.RA120.012587 PMC7535906

[79] Zhang Z, Song M, Liu X, Kang SS, Kwon I-S, Duong DM, et al. Cleavage of tau by asparagine endopeptidase mediates the neurofibrillary pathology in Alzheimer’s disease. Nat Med. 2014;20:1254–62. 25326800 10.1038/nm.3700 PMC4224595

[80] Zhang Z, Li X-G, Wang Z-H, Song M, Yu SP, Kang SS, et al. δ-Secretase-cleaved Tau stimulates Aβ production via upregulating STAT1-BACE1 signaling in Alzheimer’s disease. Mol Psychiatry. 2021;26:586–603. 30382187 10.1038/s41380-018-0286-z PMC6684859

[81] Zhang Z, Obianyo O, Dall E, Du Y, Fu H, Liu X, et al. Inhibition of delta-secretase improves cognitive functions in mouse models of Alzheimer’s disease. Nat Commun. 2017;8:14740. 28345579 10.1038/ncomms14740 PMC5378956

[82] Burke WJ, Li SW, Schmitt CA, Xia P, Chung HD, Gillespie KN. Accumulation of 3,4-dihydroxyphenylglycolaldehyde, the neurotoxic monoamine oxidase A metabolite of norepinephrine, in locus ceruleus cell bodies in Alzheimer’s disease: mechanism of neuron death. Brain Res. 1999;816:633–7. 9878889 10.1016/S0006-8993(98)01211-6

[83] Kang SS, Liu X, Ahn EH, Xiang J, Manfredsson FP, Yang X, et al. Norepinephrine metabolite DOPEGAL activates AEP and pathological Tau aggregation in locus coeruleus. J Clin Invest. 2020;130:422–37. 31793911 10.1172/JCI130513 PMC6934194

[84] Kang SS, Meng L, Zhang X, Wu Z, Mancieri A, Xie B, et al. Tau modification by the norepinephrine metabolite DOPEGAL stimulates its pathology and propagation. Nat Struct Mol Biol. 2022;29:292–305. 35332321 10.1038/s41594-022-00745-3 PMC9018606

[85] Huseby CJ, Bundschuh R, Kuret J. The role of annealing and fragmentation in human tau aggregation dynamics. J Biol Chem. 2019;294:4728–37. 30745358 10.1074/jbc.RA118.006943 PMC6442056

[86] Götz J, Chen F, van Dorpe J, Nitsch RM. Formation of neurofibrillary tangles in P301l tau transgenic mice induced by Abeta 42 fibrils. Science. 2001;293:1491–5. 11520988 10.1126/science.1062097

[87] Lewis J, Dickson DW, Lin WL, Chisholm L, Corral A, Jones G, et al. Enhanced neurofibrillary degeneration in transgenic mice expressing mutant tau and APP. Science. 2001;293:1487–91. 11520987 10.1126/science.1058189

[88] Sepulcre J, Schultz AP, Sabuncu M, Gomez-Isla T, Chhatwal J, Becker A, et al. In vivo tau, amyloid, and gray matter profiles in the aging brain. J Neurosci. 2016;36:7364–74. 27413148 10.1523/JNEUROSCI.0639-16.2016 PMC4945661

[89] Sanchez JS, Becker JA, Jacobs HIL, Hanseeuw BJ, Jiang S, Schultz AP, et al. The cortical origin and initial spread of medial temporal tauopathy in Alzheimer’s disease assessed with positron emission tomography. Sci Transl Med. 2021;13:eabc0655. 33472953 10.1126/scitranslmed.abc0655 PMC7978042

[90] Arima K, Mizutani T, Alim MA, Tonozuka-Uehara H, Izumiyama Y, Hirai S, et al. NACP/alpha-synuclein and tau constitute two distinctive subsets of filaments in the same neuronal inclusions in brains from a family of parkinsonism and dementia with Lewy bodies: double-immunolabeling fluorescence and electron microscopic studies. Acta Neuropathol. 2000;100:115–21. 10963357 10.1007/s004010050002

[91] Colom-Cadena M, Gelpi E, Charif S, Belbin O, Blesa R, Martí MJ, et al. Confluence of α-synuclein, tau, and β-amyloid pathologies in dementia with Lewy bodies. J Neuropathol Exp Neurol. 2013;72:1203–12. 24226269 10.1097/NEN.0000000000000018

[92] Ishizawa T, Mattila P, Davies P, Wang D, Dickson DW. Colocalization of tau and alpha-synuclein epitopes in Lewy bodies. J Neuropathol Exp Neurol. 2003;62:389–97. 12722831 10.1093/jnen/62.4.389

[93] Jensen PH, Hager H, Nielsen MS, Hojrup P, Gliemann J, Jakes R. alpha-synuclein binds to Tau and stimulates the protein kinase A-catalyzed tau phosphorylation of serine residues 262 and 356. J Biol Chem. 1999;274:25481–9. 10464279 10.1074/jbc.274.36.25481

[94] Giasson BI, Forman MS, Higuchi M, Golbe LI, Graves CL, Kotzbauer PT, et al. Initiation and synergistic fibrillization of tau and alpha-synuclein. Science. 2003;300:636–40. 12714745 10.1126/science.1082324

[95] Bassil F, Meymand ES, Brown HJ, Xu H, Cox TO, Pattabhiraman S, et al. alpha-Synuclein modulates tau spreading in mouse brains. J Exp Med. 2021;218(1):e20192193. 33091110 10.1084/jem.20192193 PMC7588140

[96] Waxman EA, Giasson BI. Induction of intracellular tau aggregation is promoted by α-synuclein seeds and provides novel insights into the hyperphosphorylation of tau. J Neurosci. 2011;31:7604–18. 21613474 10.1523/JNEUROSCI.0297-11.2011 PMC3122484

[97] Guo JL, Covell DJ, Daniels JP, Iba M, Stieber A, Zhang B, et al. Distinct α-synuclein strains differentially promote tau inclusions in neurons. Cell. 2013;154:103–17. 23827677 10.1016/j.cell.2013.05.057 PMC3820001

[98] Luk KC, Kehm VM, Zhang B, O’Brien P, Trojanowski JQ, Lee VMY. Intracerebral inoculation of pathological α-synuclein initiates a rapidly progressive neurodegenerative α-synucleinopathy in mice. J Exp Med. 2012;209:975–86. 22508839 10.1084/jem.20112457 PMC3348112

[99] Pan L, Li C, Meng L, Tian Y, He M, Yuan X, et al. Tau accelerates α-synuclein aggregation and spreading in Parkinson’s disease. Brain. 2022;145:3454–71. 35552614 10.1093/brain/awac171

[100] Biessels GJ, Staekenborg S, Brunner E, Brayne C, Scheltens P. Risk of dementia in diabetes mellitus: a systematic review. Lancet Neurol. 2006;5:64–74. 16361024 10.1016/S1474-4422(05)70284-2

[101] Westermark P, Andersson A, Westermark GT. Islet amyloid polypeptide, islet amyloid, and diabetes mellitus. Physiol Rev. 2011;91:795–826. 21742788 10.1152/physrev.00042.2009

[102] Zhang G, Meng L, Wang Z, Peng Q, Chen G, Xiong J, et al. Islet amyloid polypeptide cross-seeds tau and drives the neurofibrillary pathology in Alzheimer’s disease. Mol Neurodegener. 2022;17:12. 35093145 10.1186/s13024-022-00518-y PMC8800231

[103] Sanders DW, Kaufman SK, Holmes BB, Diamond MI. Prions and protein assemblies that convey biological information in health and disease. Neuron. 2016;89:433–48. 26844828 10.1016/j.neuron.2016.01.026 PMC4748384

[104] Clavaguera F, Bolmont T, Crowther RA, Abramowski D, Frank S, Probst A, et al. Transmission and spreading of tauopathy in transgenic mouse brain. Nat Cell Biol. 2009;11:909–13. 19503072 10.1038/ncb1901 PMC2726961

[105] Sanders DW, Kaufman SK, DeVos SL, Sharma AM, Mirbaha H, Li A, et al. Distinct tau prion strains propagate in cells and mice and define different tauopathies. Neuron. 2014;82:1271–88. 24857020 10.1016/j.neuron.2014.04.047 PMC4171396

[106] Frost B, Jacks RL, Diamond MI. Propagation of tau misfolding from the outside to the inside of a cell. J Biol Chem. 2009;284:12845–52. 19282288 10.1074/jbc.M808759200 PMC2676015

[107] Scheres SH, Zhang W, Falcon B, Goedert M. Cryo-EM structures of tau filaments. Curr Opin Struct Biol. 2020;64:17–25. 32603876 10.1016/j.sbi.2020.05.011

[108] Scheres SHW, Ryskeldi-Falcon B, Goedert M. Molecular pathology of neurodegenerative diseases by cryo-EM of amyloids. Nature. 2023;621:701–10. 37758888 10.1038/s41586-023-06437-2

[109] Wischik CM, Novak M, Edwards PC, Klug A, Tichelaar W, Crowther RA. Structural characterization of the core of the paired helical filament of Alzheimer disease. Proc Natl Acad Sci U S A. 1988;85:4884–8. 2455299 10.1073/pnas.85.13.4884 PMC280541

[110] Falcon B, Zhang W, Schweighauser M, Murzin AG, Vidal R, Garringer HJ, et al. Tau filaments from multiple cases of sporadic and inherited Alzheimer’s disease adopt a common fold. Acta Neuropathol. 2018;136:699–708. 30276465 10.1007/s00401-018-1914-z PMC6208733

[111] Falcon B, Zhang W, Murzin AG, Murshudov G, Garringer HJ, Vidal R, et al. Structures of filaments from Pick’s disease reveal a novel tau protein fold. Nature. 2018;561:137–40. 30158706 10.1038/s41586-018-0454-y PMC6204212

[112] Falcon B, Zivanov J, Zhang W, Murzin AG, Garringer HJ, Vidal R, et al. Novel tau filament fold in chronic traumatic encephalopathy encloses hydrophobic molecules. Nature. 2019;568:420–3. 30894745 10.1038/s41586-019-1026-5 PMC6472968

[113] Shi Y, Zhang W, Yang Y, Murzin AG, Falcon B, Kotecha A, et al. Structure-based classification of tauopathies. Nature. 2021;598:359–63. 34588692 10.1038/s41586-021-03911-7 PMC7611841

[114] Serrano-Pozo A, Frosch MP, Masliah E, Hyman BT. Neuropathological alterations in Alzheimer disease. Cold Spring Harb Perspect Med. 2011;1:a006189. 22229116 10.1101/cshperspect.a006189 PMC3234452

[115] Braak H, Del Tredici K. The pathological process underlying Alzheimer’s disease in individuals under thirty. Acta Neuropathol. 2011;121:171–81. 21170538 10.1007/s00401-010-0789-4

[116] Ehrenberg AJ, Nguy AK, Theofilas P, Dunlop S, Suemoto CK, Di Lorenzo Alho AT, et al. Quantifying the accretion of hyperphosphorylated tau in the locus coeruleus and dorsal raphe nucleus: the pathological building blocks of early Alzheimer’s disease. Neuropathol Appl Neurobiol. 2017;43:393–408. 28117917 10.1111/nan.12387 PMC5642282

[117] Bueichekú E, Diez I, Kim C-M, Becker JA, Koops EA, Kwong K, et al. Spatiotemporal patterns of locus coeruleus integrity predict cortical tau and cognition. Nat Aging. 2024;4(5):625–37. 38664576 10.1038/s43587-024-00626-y PMC11108787

[118] Schoonhoven DN, Coomans EM, Millán AP, van Nifterick AM, Visser D, Ossenkoppele R, et al. Tau protein spreads through functionally connected neurons in Alzheimer’s disease: a combined MEG/PET study. Brain. 2023;146:4040–54. 37279597 10.1093/brain/awad189 PMC10545627

[119] Williams DR, Holton JL, Strand C, Pittman A, de Silva R, Lees AJ, et al. Pathological tau burden and distribution distinguishes progressive supranuclear palsy-parkinsonism from Richardson’s syndrome. Brain. 2007;130:1566–76. 17525140 10.1093/brain/awm104

[120] Saito Y, Ruberu NN, Sawabe M, Arai T, Tanaka N, Kakuta Y, et al. Staging of argyrophilic grains: an age-associated tauopathy. J Neuropathol Exp Neurol. 2004;63:911–8. 15453090 10.1093/jnen/63.9.911

[121] Wang Y, Balaji V, Kaniyappan S, Krüger L, Irsen S, Tepper K, et al. The release and trans-synaptic transmission of Tau via exosomes. Mol Neurodegener. 2017;12:5. 28086931 10.1186/s13024-016-0143-y PMC5237256

[122] Saman S, Kim W, Raya M, Visnick Y, Miro S, Saman S, et al. Exosome-associated tau is secreted in tauopathy models and is selectively phosphorylated in cerebrospinal fluid in early Alzheimer disease. J Biol Chem. 2012;287:3842–9. 22057275 10.1074/jbc.M111.277061 PMC3281682

[123] Fiandaca MS, Kapogiannis D, Mapstone M, Boxer A, Eitan E, Schwartz JB, et al. Identification of preclinical Alzheimer’s disease by a profile of pathogenic proteins in neurally derived blood exosomes: A case-control study. Alzheimers Dement. 2015;11:600-607.e1. 25130657 10.1016/j.jalz.2014.06.008 PMC4329112

[124] Wu JW, Herman M, Liu L, Simoes S, Acker CM, Figueroa H, et al. Small misfolded Tau species are internalized via bulk endocytosis and anterogradely and retrogradely transported in neurons. J Biol Chem. 2013;288:1856–70. 23188818 10.1074/jbc.M112.394528 PMC3548495

[125] Ruan Z, Pathak D, Venkatesan Kalavai S, Yoshii-Kitahara A, Muraoka S, Bhatt N, et al. Alzheimer’s disease brain-derived extracellular vesicles spread tau pathology in interneurons. Brain. 2021;144(1):288–309. 33246331 10.1093/brain/awaa376 PMC7880668

[126] Holmes BB, DeVos SL, Kfoury N, Li M, Jacks R, Yanamandra K, et al. Heparan sulfate proteoglycans mediate internalization and propagation of specific proteopathic seeds. Proc Natl Acad Sci U S A. 2013;110:E3138-3147. 23898162 10.1073/pnas.1301440110 PMC3746848

[127] Rauch JN, Luna G, Guzman E, Audouard M, Challis C, Sibih YE, et al. LRP1 is a master regulator of tau uptake and spread. Nature. 2020;580:381–5. 32296178 10.1038/s41586-020-2156-5 PMC7687380

[128] Evans LD, Wassmer T, Fraser G, Smith J, Perkinton M, Billinton A, et al. Extracellular monomeric and aggregated tau efficiently enter human neurons through overlapping but distinct pathways. Cell Rep. 2018;22:3612–24. 29590627 10.1016/j.celrep.2018.03.021 PMC5896171

[129] Calafate S, Flavin W, Verstreken P, Moechars D. Loss of Bin1 promotes the propagation of tau pathology. Cell Rep. 2016;17:931–40. 27760323 10.1016/j.celrep.2016.09.063

[130] Kim Y, Park H, Kim Y, Kim S-H, Lee JH, Yang H, et al. Pathogenic role of RAGE in tau transmission and memory deficits. Biol Psychiatry. 2023;93:829–41. 36759256 10.1016/j.biopsych.2022.10.015

[131] Morozova V, Cohen LS, Makki AE-H, Shur A, Pilar G, El Idrissi A, et al. Normal and pathological tau uptake mediated by m1/m3 muscarinic receptors promotes opposite neuronal changes. Front Cell Neurosci. 2019;13:403. 31555098 10.3389/fncel.2019.00403 PMC6737038

[132] Andersson CR, Falsig J, Stavenhagen JB, Christensen S, Kartberg F, Rosenqvist N, et al. Antibody-mediated clearance of tau in primary mouse microglial cultures requires Fcγ-receptor binding and functional lysosomes. Sci Rep. 2019;9:4658. 30874605 10.1038/s41598-019-41105-4 PMC6420568

[133] Asai H, Ikezu S, Tsunoda S, Medalla M, Luebke J, Haydar T, et al. Depletion of microglia and inhibition of exosome synthesis halt tau propagation. Nat Neurosci. 2015;18:1584–93. 26436904 10.1038/nn.4132 PMC4694577

[134] Bolós M, Llorens-Martín M, Jurado-Arjona J, Hernández F, Rábano A, Avila J. Direct evidence of internalization of tau by microglia in vitro and in vivo. J Alzheimers Dis. 2016;50:77–87. 26638867 10.3233/JAD-150704

[135] Zilkova M, Nolle A, Kovacech B, Kontsekova E, Weisova P, Filipcik P, et al. Humanized tau antibodies promote tau uptake by human microglia without any increase of inflammation. Acta Neuropathol Commun. 2020;8:74. 32471486 10.1186/s40478-020-00948-z PMC7257136

[136] Funk KE, Mirbaha H, Jiang H, Holtzman DM, Diamond MI. Distinct therapeutic mechanisms of tau antibodies: promoting microglial clearance versus blocking neuronal uptake. J Biol Chem. 2015;290:21652–62. 26126828 10.1074/jbc.M115.657924 PMC4571888

[137] Bolós M, Llorens-Martín M, Perea JR, Jurado-Arjona J, Rábano A, Hernández F, et al. Absence of CX3CR1 impairs the internalization of Tau by microglia. Mol Neurodegener. 2017;12:59. 28810892 10.1186/s13024-017-0200-1 PMC5558740

[138] Das R, Chinnathambi S. Microglial remodeling of actin network by Tau oligomers, via G protein-coupled purinergic receptor, P2Y12R-driven chemotaxis. Traffic. 2021;22:153–70. 33527700 10.1111/tra.12784

[139] Odfalk KF, Bieniek KF, Hopp SC. Microglia: Friend and foe in tauopathy. Prog Neurobiol. 2022;216:102306. 35714860 10.1016/j.pneurobio.2022.102306 PMC9378545

[140] Sanchez-Mejias E, Navarro V, Jimenez S, Sanchez-Mico M, Sanchez-Varo R, Nuñez-Diaz C, et al. Soluble phospho-tau from Alzheimer’s disease hippocampus drives microglial degeneration. Acta Neuropathol. 2016;132:897–916. 27743026 10.1007/s00401-016-1630-5 PMC5106501

[141] Crotti A, Sait HR, McAvoy KM, Estrada K, Ergun A, Szak S, et al. BIN1 favors the spreading of Tau via extracellular vesicles. Sci Rep. 2019;9:9477. 31263146 10.1038/s41598-019-45676-0 PMC6603165

[142] Clayton K, Delpech JC, Herron S, Iwahara N, Ericsson M, Saito T, et al. Plaque associated microglia hyper-secrete extracellular vesicles and accelerate tau propagation in a humanized APP mouse model. Mol Neurodegener. 2021;16:18. 33752701 10.1186/s13024-021-00440-9 PMC7986521

[143] Gorlovoy P, Larionov S, Pham TTH, Neumann H. Accumulation of tau induced in neurites by microglial proinflammatory mediators. FASEB J. 2009;23:2502–13. 19289607 10.1096/fj.08-123877

[144] Maphis N, Xu G, Kokiko-Cochran ON, Jiang S, Cardona A, Ransohoff RM, et al. Reactive microglia drive tau pathology and contribute to the spreading of pathological tau in the brain. Brain. 2015;138:1738–55. 25833819 10.1093/brain/awv081 PMC4542622

[145] Luo W, Liu W, Hu X, Hanna M, Caravaca A, Paul SM. Microglial internalization and degradation of pathological tau is enhanced by an anti-tau monoclonal antibody. Sci Rep. 2015;5:11161. 26057852 10.1038/srep11161 PMC4460904

[146] Majerova P, Zilkova M, Kazmerova Z, Kovac A, Paholikova K, Kovacech B, et al. Microglia display modest phagocytic capacity for extracellular tau oligomers. J Neuroinflammation. 2014;11:161. 25217135 10.1186/s12974-014-0161-z PMC4172893

[147] Liang S-Y, Wang Z-T, Tan L, Yu J-T. Tau toxicity in neurodegeneration. Mol Neurobiol. 2022;59:3617–34. 35359226 10.1007/s12035-022-02809-3

[148] Cowan CM, Mudher A. Are tau aggregates toxic or protective in tauopathies? Front Neurol. 2013;4:114. 23964266 10.3389/fneur.2013.00114 PMC3741634

[149] Wang W, Zhao F, Ma X, Perry G, Zhu X. Mitochondria dysfunction in the pathogenesis of Alzheimer’s disease: recent advances. Mol Neurodegener. 2020;15:30. 32471464 10.1186/s13024-020-00376-6 PMC7257174

[150] Sanchez-Varo R, Trujillo-Estrada L, Sanchez-Mejias E, Torres M, Baglietto-Vargas D, Moreno-Gonzalez I, et al. Abnormal accumulation of autophagic vesicles correlates with axonal and synaptic pathology in young Alzheimer’s mice hippocampus. Acta Neuropathol. 2012;123:53–70. 22020633 10.1007/s00401-011-0896-x PMC3249205

[151] Niewiadomska G, Niewiadomski W, Steczkowska M, Gasiorowska A. Tau oligomers neurotoxicity. Life (Basel). 2021;11:28. 33418848 10.3390/life11010028 PMC7824853

[152] Braak H, Braak E. Neuropathological stageing of Alzheimer-related changes. Acta Neuropathol. 1991;82:239–59. 1759558 10.1007/BF00308809

[153] Arriagada PV, Growdon JH, Hedley-Whyte ET, Hyman BT. Neurofibrillary tangles but not senile plaques parallel duration and severity of Alzheimer’s disease. Neurology. 1992;42:631–9. 1549228 10.1212/WNL.42.3.631

[154] Nagy Z, Esiri MM, Jobst KA, Morris JH, King EM, McDonald B, et al. Relative roles of plaques and tangles in the dementia of Alzheimer’s disease: correlations using three sets of neuropathological criteria. Dementia. 1995;6:21–31. 7728216 10.1159/000106918

[155] Usenovic M, Niroomand S, Drolet RE, Yao L, Gaspar RC, Hatcher NG, et al. Internalized tau oligomers cause neurodegeneration by inducing accumulation of pathogenic tau in human neurons derived from induced pluripotent stem cells. J Neurosci. 2015;35:14234–50. 26490863 10.1523/JNEUROSCI.1523-15.2015 PMC6605424

[156] Fá M, Puzzo D, Piacentini R, Staniszewski A, Zhang H, Baltrons MA, et al. Extracellular tau oligomers produce an immediate impairment of LTP and memory. Sci Rep. 2016;6:19393. 26786552 10.1038/srep19393 PMC4726138

[157] Lasagna-Reeves CA, Castillo-Carranza DL, Sengupta U, Clos AL, Jackson GR, Kayed R. Tau oligomers impair memory and induce synaptic and mitochondrial dysfunction in wild-type mice. Mol Neurodegener. 2011;6:39. 21645391 10.1186/1750-1326-6-39 PMC3224595

[158] O’Leary JC, Li Q, Marinec P, Blair LJ, Congdon EE, Johnson AG, et al. Phenothiazine-mediated rescue of cognition in tau transgenic mice requires neuroprotection and reduced soluble tau burden. Mol Neurodegener. 2010;5:45. 21040568 10.1186/1750-1326-5-45 PMC2989315

[159] Sun X, Eastman G, Shi Y, Saibaba S, Oliveira AK, Lukens JR, et al. Structural and functional damage to neuronal nuclei caused by extracellular tau oligomers. Alzheimers Dement. 2024;20:1656–70. 38069673 10.1002/alz.13535 PMC10947977

[160] Clinton LK, Blurton-Jones M, Myczek K, Trojanowski JQ, LaFerla FM. Synergistic Interactions between Abeta, tau, and alpha-synuclein: acceleration of neuropathology and cognitive decline. J Neurosci. 2010;30:7281–9. 20505094 10.1523/JNEUROSCI.0490-10.2010 PMC3308018

[161] Torres-Garcia L, Domingues PJM, Brandi E, Haikal C, Mudannayake JM, Brás IC, et al. Monitoring the interactions between alpha-synuclein and Tau in vitro and in vivo using bimolecular fluorescence complementation. Sci Rep. 2022;12:2987. 35194057 10.1038/s41598-022-06846-9 PMC8863885

[162] Bassil F, Brown HJ, Pattabhiraman S, Iwasyk JE, Maghames CM, Meymand ES, et al. Amyloid-beta (Aβ) plaques promote seeding and spreading of alpha-synuclein and tau in a mouse model of lewy body disorders with aβ pathology. Neuron. 2020;105:260-275.e6. 31759806 10.1016/j.neuron.2019.10.010 PMC6981053

[163] Schindler SE, Jasielec MS, Weng H, Hassenstab JJ, Grober E, McCue LM, et al. Neuropsychological measures that detect early impairment and decline in preclinical Alzheimer disease. Neurobiol Aging. 2017;56:25–32. 28482211 10.1016/j.neurobiolaging.2017.04.004 PMC5505233

[164] Clark LR, Berman SE, Norton D, Koscik RL, Jonaitis E, Blennow K, et al. Age-accelerated cognitive decline in asymptomatic adults with CSF β-amyloid. Neurology. 2018;90:e1306-15. 29523644 10.1212/WNL.0000000000005291 PMC5894934

[165] Hanseeuw BJ, Betensky RA, Schultz AP, Papp KV, Mormino EC, Sepulcre J, et al. Fluorodeoxyglucose metabolism associated with tau-amyloid interaction predicts memory decline. Ann Neurol. 2017;81:583–96. 28253546 10.1002/ana.24910 PMC5404378

[166] Zhang H, Wei W, Zhao M, Ma L, Jiang X, Pei H, et al. Interaction between Aβ and Tau in the Pathogenesis of Alzheimer’s Disease. Int J Biol Sci. 2021;17:2181–92. 34239348 10.7150/ijbs.57078 PMC8241728

[167] Campion D, Pottier C, Nicolas G, Le Guennec K, Rovelet-Lecrux A. Alzheimer disease: modeling an Aβ-centered biological network. Mol Psychiatry. 2016;21:861–71. 27021818 10.1038/mp.2016.38

[168] Li W, Li J-Y. Overlaps and divergences between tauopathies and synucleinopathies: a duet of neurodegeneration. Transl Neurodegener. 2024;13:16. 38528629 10.1186/s40035-024-00407-y PMC10964635

[169] Castillo-Carranza DL, Guerrero-Muñoz MJ, Sengupta U, Gerson JE, Kayed R. α-Synuclein oligomers induce a unique toxic tau strain. Biol Psychiatry. 2018;84:499–508. 29478699 10.1016/j.biopsych.2017.12.018 PMC6201292

[170] Williams T, Sorrentino Z, Weinrich M, Giasson BI, Chakrabarty P. Differential cross-seeding properties of tau and α-synuclein in mouse models of tauopathy and synucleinopathy. Brain Commun. 2020;2:fcaa090. 33094280 10.1093/braincomms/fcaa090 PMC7567170

[171] Xia Y, Prokop S, Giasson BI. “Don’t Phos Over Tau”: recent developments in clinical biomarkers and therapies targeting tau phosphorylation in Alzheimer’s disease and other tauopathies. Mol Neurodegener. 2021;16:37. 34090488 10.1186/s13024-021-00460-5 PMC8180161

[172] Caballero B, Bourdenx M, Luengo E, Diaz A, Sohn PD, Chen X, et al. Acetylated tau inhibits chaperone-mediated autophagy and promotes tau pathology propagation in mice. Nat Commun. 2021;12:2238. 33854069 10.1038/s41467-021-22501-9 PMC8047017

[173] Alquezar C, Schoch KM, Geier EG, Ramos EM, Scrivo A, Li KH, et al. TSC1 loss increases risk for tauopathy by inducing tau acetylation and preventing tau clearance via chaperone-mediated autophagy. Sci Adv. 2021;7:eabg3897. 34739309 10.1126/sciadv.abg3897 PMC8570595

[174] Alquezar C, Arya S, Kao AW. Tau post-translational modifications: dynamic transformers of tau function, degradation, and aggregation. Front Neurol. 2020;11:595532. 33488497 10.3389/fneur.2020.595532 PMC7817643

[175] Quinn JP, Corbett NJ, Kellett KAB, Hooper NM. Tau proteolysis in the pathogenesis of tauopathies: neurotoxic fragments and novel biomarkers. J Alzheimers Dis. 2018;63:13–33. 29630551 10.3233/JAD-170959 PMC5900574

[176] Ossenkoppele R, van der Kant R, Hansson O. Tau biomarkers in Alzheimer’s disease: towards implementation in clinical practice and trials. Lancet Neurol. 2022;21:726–34. 35643092 10.1016/S1474-4422(22)00168-5

[177] Barthélemy NR, Bateman RJ, Hirtz C, Marin P, Becher F, Sato C, et al. Cerebrospinal fluid phospho-tau T217 outperforms T181 as a biomarker for the differential diagnosis of Alzheimer’s disease and PET amyloid-positive patient identification. Alzheimers Res Ther. 2020;12:26. 32183883 10.1186/s13195-020-00596-4 PMC7079453

[178] Mielke MM, Aakre JA, Algeciras-Schimnich A, Proctor NK, Machulda MM, Eichenlaub U, et al. Comparison of CSF phosphorylated tau 181 and 217 for cognitive decline. Alzheimers Dement. 2022;18:602–11. 34310832 10.1002/alz.12415 PMC8789950

[179] Janelidze S, Stomrud E, Smith R, Palmqvist S, Mattsson N, Airey DC, et al. Cerebrospinal fluid p-tau217 performs better than p-tau181 as a biomarker of Alzheimer’s disease. Nat Commun. 2020;11:1683. 32246036 10.1038/s41467-020-15436-0 PMC7125218

[180] Lantero-Rodriguez J, Montoliu-Gaya L, Benedet AL, Vrillon A, Dumurgier J, Cognat E, et al. CSF p-tau205: a biomarker of tau pathology inAlzheimer’s disease. Acta Neuropathol. 2024;147:12. 38184490 10.1007/s00401-023-02659-w PMC10771353

[181] Horie K, Salvadó G, Barthélemy NR, Janelidze S, Li Y, He Y, et al. CSF MTBR-tau243 is a specific biomarker of tau tangle pathology in Alzheimer’s disease. Nat Med. 2023;29:1954–63. 37443334 10.1038/s41591-023-02443-z PMC10427417

[182] Blennow K, Chen C, Cicognola C, Wildsmith KR, Manser PT, Bohorquez SMS, et al. Cerebrospinal fluid tau fragment correlates with tau PET: a candidate biomarker for tangle pathology. Brain. 2020;143:650–60. 31834365 10.1093/brain/awz346 PMC7009597

[183] Simrén J, Brum WS, Ashton NJ, Benedet AL, Karikari TK, Kvartsberg H, et al. CSF tau368/total-tau ratio reflects cognitive performance and neocortical tau better compared to p-tau181 and p-tau217 in cognitively impaired individuals. Alzheimers Res Ther. 2022;14:192. 36544221 10.1186/s13195-022-01142-0 PMC9773470

[184] Wang X, Bakulski KM, Karvonen-Gutierrez CA, Park SK, Morgan D, Albin RL, et al. Blood-based biomarkers for Alzheimer’s disease and cognitive function from mid- to late life. Alzheimers Dement. 2024;20:1807–14. 38126555 10.1002/alz.13583 PMC10984504

[185] Karikari TK, Pascoal TA, Ashton NJ, Janelidze S, Benedet AL, Rodriguez JL, et al. Blood phosphorylated tau 181 as a biomarker for Alzheimer’s disease: a diagnostic performance and prediction modelling study using data from four prospective cohorts. Lancet Neurol. 2020;19:422–33. 32333900 10.1016/S1474-4422(20)30071-5

[186] Brickman AM, Manly JJ, Honig LS, Sanchez D, Reyes-Dumeyer D, Lantigua RA, et al. Plasma p-tau181, p-tau217, and other blood-based Alzheimer’s disease biomarkers in a multi-ethnic, community study. Alzheimers Dement. 2021;17:1353–64. 33580742 10.1002/alz.12301 PMC8451860

[187] Ashton NJ, Pascoal TA, Karikari TK, Benedet AL, Lantero-Rodriguez J, Brinkmalm G, et al. Plasma p-tau231: a new biomarker for incipient Alzheimer’s disease pathology. Acta Neuropathol. 2021;141:709–24. 33585983 10.1007/s00401-021-02275-6 PMC8043944

[188] Mattsson-Carlgren N, Janelidze S, Palmqvist S, Cullen N, Svenningsson AL, Strandberg O, et al. Longitudinal plasma p-tau217 is increased in early stages of Alzheimer’s disease. Brain. 2020;143:3234–41. 33068398 10.1093/brain/awaa286 PMC7719022

[189] Mattsson-Carlgren N, Janelidze S, Bateman RJ, Smith R, Stomrud E, Serrano GE, et al. Soluble P-tau217 reflects amyloid and tau pathology and mediates the association of amyloid with tau. EMBO Mol Med. 2021;13: e14022. 33949133 10.15252/emmm.202114022 PMC8185545

[190] Palmqvist S, Janelidze S, Quiroz YT, Zetterberg H, Lopera F, Stomrud E, et al. Discriminative accuracy of plasma phospho-tau217 for Alzheimer disease vs other neurodegenerative disorders. JAMA. 2020;324:772–81. 32722745 10.1001/jama.2020.12134 PMC7388060

[191] Ferreira PCL, Therriault J, Tissot C, Ferrari-Souza JP, Benedet AL, Povala G, et al. Plasma p-tau231 and p-tau217 inform on tau tangles aggregation in cognitively impaired individuals. Alzheimers Dement. 2023;19:4463–74. 37534889 10.1002/alz.13393 PMC10592380

[192] Jack CR, Wiste HJ, Algeciras-Schimnich A, Weigand SD, Figdore DJ, Lowe VJ, et al. Comparison of plasma biomarkers and amyloid PET for predicting memory decline in cognitively unimpaired individuals. Alzheimers Dement. 2024;20:2143–54. 38265198 10.1002/alz.13651 PMC10984437

[193] Thijssen EH, La Joie R, Strom A, Fonseca C, Iaccarino L, Wolf A, et al. Plasma phosphorylated tau 217 and phosphorylated tau 181 as biomarkers in Alzheimer’s disease and frontotemporal lobar degeneration: a retrospective diagnostic performance study. Lancet Neurol. 2021;20:739–52. 34418401 10.1016/S1474-4422(21)00214-3 PMC8711249

[194] Hansson O, Edelmayer RM, Boxer AL, Carrillo MC, Mielke MM, Rabinovici GD, et al. The Alzheimer’s Association appropriate use recommendations for blood biomarkers in Alzheimer’s disease. Alzheimers Dement. 2022;18:2669–86. 35908251 10.1002/alz.12756 PMC10087669

[195] Barthélemy NR, Salvadó G, Schindler SE, He Y, Janelidze S, Collij LE, et al. Highly accurate blood test for Alzheimer’s disease is similar or superior to clinical cerebrospinal fluid tests. Nat Med. 2024;30:1085–95. 38382645 10.1038/s41591-024-02869-z PMC11031399

[196] Ashton NJ, Brum WS, Di Molfetta G, Benedet AL, Arslan B, Jonaitis E, et al. Diagnostic accuracy of a plasma phosphorylated tau 217 immunoassay for Alzheimer disease pathology. JAMA Neurol. 2024;81:255–63. 38252443 10.1001/jamaneurol.2023.5319 PMC10804282

[197] Kac PR, González-Ortiz F, Emeršič A, Dulewicz M, Koutarapu S, Turton M, et al. Plasma p-tau212 antemortem diagnostic performance and prediction of autopsy verification of Alzheimer’s disease neuropathology. Nat Commun. 2024;15:2615. 38521766 10.1038/s41467-024-46876-7 PMC10960791

[198] Gonzalez-Ortiz F, Kirsebom B-E, Contador J, Tanley JE, Selnes P, Gísladóttir B, et al. Plasma brain-derived tau is an amyloid-associated neurodegeneration biomarker in Alzheimer’s disease. Nat Commun. 2024;15:2908. 38575616 10.1038/s41467-024-47286-5 PMC10995141

[199] Cassinelli Petersen G, Roytman M, Chiang GC, Li Y, Gordon ML, Franceschi AM. Overview of tau PET molecular imaging. Curr Opin Neurol. 2022;35:230–9. 35191407 10.1097/WCO.0000000000001035 PMC9011369

[200] Lagarde J, Olivieri P, Tonietto M, Tissot C, Rivals I, Gervais P, et al. Tau-PET imaging predicts cognitive decline and brain atrophy progression in early Alzheimer’s disease. J Neurol Neurosurg Psychiatry. 2022;93:459–67. 35228270 10.1136/jnnp-2021-328623

[201] Beyer L, Nitschmann A, Barthel H, van Eimeren T, Unterrainer M, Sauerbeck J, et al. Early-phase [^18^F]PI-2620 tau-PET imaging as a surrogate marker of neuronal injury. Eur J Nucl Med Mol Imaging. 2020;47:2911–22. 10.1007/s00259-020-04788-w PMC7567714 32318783

[202] Aguero C, Dhaynaut M, Amaral AC, Moon S-H, Neelamegam R, Scapellato M, et al. Head-to-head comparison of [^18^F]-Flortaucipir, [^18^F]-MK-6240 and [^18^F]-PI-2620 postmortem binding across the spectrum of neurodegenerative diseases. Acta Neuropathol. 2024;147:25. 10.1007/s00401-023-02672-z PMC10822013 38280071

[203] Xu H, Rösler TW, Carlsson T, de Andrade A, Fiala O, Hollerhage M, et al. Tau silencing by siRNA in the P301S mouse model of tauopathy. Curr Gene Ther. 2014;14:343–51. 25687501 10.2174/156652321405140926160602

[204] DeVos SL, Miller RL, Schoch KM, Holmes BB, Kebodeaux CS, Wegener AJ, et al. Tau reduction prevents neuronal loss and reverses pathological tau deposition and seeding in mice with tauopathy. Sci Transl Med. 2017;9:eaag0481. 28123067 10.1126/scitranslmed.aag0481 PMC5792300

[205] Mummery CJ, Börjesson-Hanson A, Blackburn DJ, Vijverberg EGB, De Deyn PP, Ducharme S, et al. Tau-targeting antisense oligonucleotide MAPTRx in mild Alzheimer’s disease: a phase 1b, randomized, placebo-controlled trial. Nat Med. 2023;29:1437–47. 37095250 10.1038/s41591-023-02326-3 PMC10287562

[206] Soeda Y, Takashima A. New insights into drug discovery targeting tau protein. Front Mol Neurosci. 2020;13:590896. 33343298 10.3389/fnmol.2020.590896 PMC7744460

[207] Wischik CM, Edwards PC, Lai RY, Roth M, Harrington CR. Selective inhibition of Alzheimer disease-like tau aggregation by phenothiazines. Proc Natl Acad Sci U S A. 1996;93:11213–8. 8855335 10.1073/pnas.93.20.11213 PMC38310

[208] Akoury E, Pickhardt M, Gajda M, Biernat J, Mandelkow E, Zweckstetter M. Mechanistic basis of phenothiazine-driven inhibition of Tau aggregation. Angew Chem Int Ed Engl. 2013;52:3511–5. 23401175 10.1002/anie.201208290

[209] Liu Y, Tan Y, Cheng G, Ni Y, Xie A, Zhu X, et al. Customized intranasal hydrogel delivering methylene blue ameliorates cognitive dysfunction against Alzheimer’s disease. Adv Mater. 2024;36(19):e2307081. 38395039 10.1002/adma.202307081

[210] Hu S, Maiti P, Ma Q, Zuo X, Jones MR, Cole GM, et al. Clinical development of curcumin in neurodegenerative disease. Expert Rev Neurother. 2015;15:629–37. 26035622 10.1586/14737175.2015.1044981 PMC6800094

[211] Wang B, Pan X, Teng I-T, Li X, Kobeissy F, Wu Z-Y, et al. Functional selection of tau oligomerization-inhibiting aptamers. Angew Chem Int Ed Engl. 2024;63:e202402007. 38407551 10.1002/anie.202402007 PMC12498309

[212] Chai K, Yang J, Tu Y, Wu J, Fang K, Shi S, et al. Molecular deformation is a key factor in screening aggregation inhibitor for intrinsically disordered protein tau. ACS Cent Sci. 2024;10:717–28. 38559297 10.1021/acscentsci.3c01196 PMC10979476

[213] Lokireddy S, Kukushkin NV, Goldberg AL. cAMP-induced phosphorylation of 26S proteasomes on Rpn6/PSMD11 enhances their activity and the degradation of misfolded proteins. Proc Natl Acad Sci U S A. 2015;112:E7176-7185. 26669444 10.1073/pnas.1522332112 PMC4702992

[214] Prickaerts J, Heckman PRA, Blokland A. Investigational phosphodiesterase inhibitors in phase I and phase II clinical trials for Alzheimer’s disease. Expert Opin Investig Drugs. 2017;26:1033–48. 28772081 10.1080/13543784.2017.1364360

[215] Gallardo G, Wong CH, Ricardez SM, Mann CN, Lin KH, Leyns CEG, et al. Targeting tauopathy with engineered tau-degrading intrabodies. Mol Neurodegener. 2019;14:38. 31640765 10.1186/s13024-019-0340-6 PMC6805661

[216] Wang W, Zhou Q, Jiang T, Li S, Ye J, Zheng J, et al. A novel small-molecule PROTAC selectively promotes tau clearance to improve cognitive functions in Alzheimer-like models. Theranostics. 2021;11:5279–95. 33859747 10.7150/thno.55680 PMC8039949

[217] Bhatia S, Singh M, Singh T, Singh V. Scrutinizing the therapeutic potential of PROTACs in the management of Alzheimer’s disease. Neurochem Res. 2023;48:13–25. 35987974 10.1007/s11064-022-03722-w

[218] Ahn G, Banik SM, Miller CL, Riley NM, Cochran JR, Bertozzi CR. LYTACs that engage the asialoglycoprotein receptor for targeted protein degradation. Nat Chem Biol. 2021;17:937–46. 33767387 10.1038/s41589-021-00770-1 PMC8387313

[219] Cotton AD, Nguyen DP, Gramespacher JA, Seiple IB, Wells JA. Development of antibody-based PROTACs for the degradation of the cell-surface immune checkpoint protein PD-L1. J Am Chem Soc. 2021;143:593–8. 33395526 10.1021/jacs.0c10008 PMC8154509

[220] Takahashi D, Arimoto H. Targeting selective autophagy by AUTAC degraders. Autophagy. 2020;16:765–6. 31958028 10.1080/15548627.2020.1718362 PMC7138220

[221] Xu L, Wu X, Zhao S, Hu H, Wang S, Zhang Y, et al. Harnessing nanochaperone-mediated autophagy for selective clearance of pathogenic tau protein in Alzheimer’s disease. Adv Mater. 2024;e2313869. 10.1002/adma.202313869. 10.1002/adma.202313869 38688523

[222] Nobuhara CK, DeVos SL, Commins C, Wegmann S, Moore BD, Roe AD, et al. Tau antibody targeting pathological species blocks neuronal uptake and interneuron propagation of tau in vitro. Am J Pathol. 2017;187:1399–412. 28408124 10.1016/j.ajpath.2017.01.022 PMC5455060

[223] Noble W, Planel E, Zehr C, Olm V, Meyerson J, Suleman F, et al. Inhibition of glycogen synthase kinase-3 by lithium correlates with reduced tauopathy and degeneration in vivo. Proc Natl Acad Sci U S A. 2005;102:6990–5. 15867159 10.1073/pnas.0500466102 PMC1088065

[224] Nakashima H, Ishihara T, Suguimoto P, Yokota O, Oshima E, Kugo A, et al. Chronic lithium treatment decreases tau lesions by promoting ubiquitination in a mouse model of tauopathies. Acta Neuropathol. 2005;110:547–56. 16228182 10.1007/s00401-005-1087-4

[225] Le Corre S, Klafki HW, Plesnila N, Hübinger G, Obermeier A, Sahagún H, et al. An inhibitor of tau hyperphosphorylation prevents severe motor impairments in tau transgenic mice. Proc Natl Acad Sci U S A. 2006;103:9673–8. 16769887 10.1073/pnas.0602913103 PMC1480465

[226] Taylor LW, Simzer EM, Pimblett C, Lacey-Solymar OTT, McGeachan RI, Meftah S, et al. p-tau Ser356 is associated with Alzheimer’s disease pathology and is lowered in brain slice cultures using the NUAK inhibitor WZ4003. Acta Neuropathol. 2024;147:7. 38175261 10.1007/s00401-023-02667-w PMC10766794

[227] Singulani MP, Ferreira AFF, Figueroa PS, Cuyul-Vásquez I, Talib LL, Britto LR, et al. Lithium and disease modification: A systematic review and meta-analysis in Alzheimer’s and Parkinson’s disease. Ageing Res Rev. 2024;95:102231. 38364914 10.1016/j.arr.2024.102231

[228] Zhang X, Heng X, Li T, Li L, Yang D, Zhang X, et al. Long-term treatment with lithium alleviates memory deficits and reduces amyloid-β production in an aged Alzheimer’s disease transgenic mouse model. J Alzheimers Dis. 2011;24:739–49. 21321394 10.3233/JAD-2011-101875

[229] Lei P, Ayton S, Appukuttan AT, Moon S, Duce JA, Volitakis I, et al. Lithium suppression of tau induces brain iron accumulation and neurodegeneration. Mol Psychiatry. 2017;22:396–406. 27400857 10.1038/mp.2016.96

[230] Hampel H, Ewers M, Bürger K, Annas P, Mörtberg A, Bogstedt A, et al. Lithium trial in Alzheimer’s disease: a randomized, single-blind, placebo-controlled, multicenter 10-week study. J Clin Psychiatry. 2009;70:922–31. 19573486 10.4088/JCP.08m04606

[231] Rueli RHLH, Torres DJ, Dewing AST, Kiyohara AC, Barayuga SM, Bellinger MT, et al. Selenoprotein S reduces endoplasmic reticulum stress-induced phosphorylation of tau: potential role in selenate mitigation of tau pathology. J Alzheimers Dis. 2017;55:749–62. 27802219 10.3233/JAD-151208 PMC5893862

[232] van Eersel J, Ke YD, Liu X, Delerue F, Kril JJ, Götz J, et al. Sodium selenate mitigates tau pathology, neurodegeneration, and functional deficits in Alzheimer’s disease models. Proc Natl Acad Sci U S A. 2010;107:13888–93. 20643941 10.1073/pnas.1009038107 PMC2922247

[233] Min S-W, Chen X, Tracy TE, Li Y, Zhou Y, Wang C, et al. Critical role of acetylation in tau-mediated neurodegeneration and cognitive deficits. Nat Med. 2015;21:1154–62. 26390242 10.1038/nm.3951 PMC4598295

[234] VandeVrede L, Dale ML, Fields S, Frank M, Hare E, Heuer HW, et al. Open-label phase 1 futility studies of salsalate and young plasma in progressive supranuclear palsy. Mov Disord Clin Pract. 2020;7:440–7. 32373661 10.1002/mdc3.12940 PMC7197321

[235] Noble W, Garwood C, Stephenson J, Kinsey AM, Hanger DP, Anderton BH. Minocycline reduces the development of abnormal tau species in models of Alzheimer’s disease. FASEB J. 2009;23:739–50. 19001528 10.1096/fj.08-113795

[236] Tan M-S, Liu Y, Hu H, Tan C-C, Tan L. Inhibition of caspase-1 ameliorates tauopathy and rescues cognitive impairment in SAMP8 mice. Metab Brain Dis. 2022;37:1197–205. 35143023 10.1007/s11011-022-00914-9

[237] Flores J, Noël A, Foveau B, Beauchet O, LeBlanc AC. Pre-symptomatic Caspase-1 inhibitor delays cognitive decline in a mouse model of Alzheimer disease and aging. Nat Commun. 2020;11:4571. 32917871 10.1038/s41467-020-18405-9 PMC7486940

[238] Rosenmann H, Grigoriadis N, Karussis D, Boimel M, Touloumi O, Ovadia H, et al. Tauopathy-like abnormalities and neurologic deficits in mice immunized with neuronal tau protein. Arch Neurol. 2006;63:1459–67. 17030663 10.1001/archneur.63.10.1459

[239] Novak P, Schmidt R, Kontsekova E, Zilka N, Kovacech B, Skrabana R, et al. Safety and immunogenicity of the tau vaccine AADvac1 in patients with Alzheimer’s disease: a randomised, double-blind, placebo-controlled, phase 1 trial. Lancet Neurol. 2017;16:123–34. 27955995 10.1016/S1474-4422(16)30331-3

[240] Novak P, Kovacech B, Katina S, Schmidt R, Scheltens P, Kontsekova E, et al. ADAMANT: a placebo-controlled randomized phase 2 study of AADvac1, an active immunotherapy against pathological tau in Alzheimer’s disease. Nat Aging. 2021;1:521–34. 37117834 10.1038/s43587-021-00070-2

[241] Novak P, Schmidt R, Kontsekova E, Kovacech B, Smolek T, Katina S, et al. FUNDAMANT: an interventional 72-week phase 1 follow-up study of AADvac1, an active immunotherapy against tau protein pathology in Alzheimer’s disease. Alzheimers Res Ther. 2018;10:108. 30355322 10.1186/s13195-018-0436-1 PMC6201586

[242] Novak P, Kontsekova E, Zilka N, Novak M. Ten years of tau-targeted immunotherapy: the path walked and the roads ahead. Front Neurosci. 2018;12:798. 30450030 10.3389/fnins.2018.00798 PMC6224648

[243] Congdon EE, Ji C, Tetlow AM, Jiang Y, Sigurdsson EM. Tau-targeting therapies for Alzheimer disease: current status and future directions. Nat Rev Neurol. 2023;19:715–36. 37875627 10.1038/s41582-023-00883-2 PMC10965012

[244] Congdon EE, Gu J, Sait HBR, Sigurdsson EM. Antibody uptake into neurons occurs primarily via clathrin-dependent Fcγ receptor endocytosis and is a prerequisite for acute tau protein clearance. J Biol Chem. 2013;288:35452–65. 24163366 10.1074/jbc.M113.491001 PMC3853292

[245] Shamir DB, Rosenqvist N, Rasool S, Pedersen JT, Sigurdsson EM. Internalization of tau antibody and pathological tau protein detected with a flow cytometry multiplexing approach. Alzheimers Dement. 2016;12:1098–107. 27016263 10.1016/j.jalz.2016.01.013 PMC5383206

[246] Teng E, Manser PT, Pickthorn K, Brunstein F, Blendstrup M, Sanabria Bohorquez S, et al. Safety and efficacy of semorinemab in individuals with prodromal to mild alzheimer disease: a randomized clinical trial. JAMA Neurol. 2022;79:758–67. 35696185 10.1001/jamaneurol.2022.1375 PMC9194753

[247] Guo X, Yan L, Zhang D, Zhao Y. Passive immunotherapy for Alzheimer’s disease. Ageing Res Rev. 2024;94:102192. 38219962 10.1016/j.arr.2024.102192

[248] Liu H, Mei F, Ye R, Han X, Wang S, Ding Y, et al. APOE3ch alleviates Aβ and tau pathology and neurodegeneration in the human APPNL-G-F cerebral organoid model of Alzheimer’s disease. Cell Res. 2024;34(6):451–4. 38609581 10.1038/s41422-024-00957-w PMC11143179

[249] Parra Bravo C, Giani AM, Madero-Perez J, Zhao Z, Wan Y, Samelson AJ, et al. Human iPSC 4R tauopathy model uncovers modifiers of tau propagation. Cell. 2024;S0092–8674(24):00306–4. 10.1016/j.cell.2024.03.015 PMC11365117 38582079

